# FAF-Drugs2: Free ADME/tox filtering tool to assist drug discovery and chemical biology projects

**DOI:** 10.1186/1471-2105-9-396

**Published:** 2008-09-24

**Authors:** David Lagorce, Olivier Sperandio, Hervé Galons, Maria A Miteva, Bruno O Villoutreix

**Affiliations:** 1 INSERM U648, MTi team, Paris Descartes University, Paris Diderot University, Paris, France; 2 INSERM U648, Chemistry team, Paris Descartes University, Paris, France

## Abstract

**Background:**

Drug discovery and chemical biology are exceedingly complex and demanding enterprises. In recent years there are been increasing awareness about the importance of predicting/optimizing the absorption, distribution, metabolism, excretion and toxicity (ADMET) properties of small chemical compounds along the search process rather than at the final stages. Fast methods for evaluating ADMET properties of small molecules often involve applying a set of simple empirical rules (educated guesses) and as such, compound collections' property profiling can be performed *in silico*. Clearly, these rules cannot assess the full complexity of the human body but can provide valuable information and assist decision-making.

**Results:**

This paper presents FAF-Drugs2, a free adaptable tool for ADMET filtering of electronic compound collections. FAF-Drugs2 is a command line utility program (e.g., written in Python) based on the open source chemistry toolkit OpenBabel, which performs various physicochemical calculations, identifies key functional groups, some toxic and unstable molecules/functional groups. In addition to filtered collections, FAF-Drugs2 can provide, via Gnuplot, several distribution diagrams of major physicochemical properties of the screened compound libraries.

**Conclusion:**

We have developed FAF-Drugs2 to facilitate compound collection preparation, prior to (or after) experimental screening or virtual screening computations. Users can select to apply various filtering thresholds and add rules as needed for a given project. As it stands, FAF-Drugs2 implements numerous filtering rules (23 physicochemical rules and 204 substructure searching rules) that can be easily tuned.

## Background

Hit/lead compounds can be identified either through high-(medium) throughput screening approaches and/or using virtual screening computations. In all situations, a compound collection is screened with the goal of finding molecules that could enter the drug discovery process or that could help to explore molecular mechanisms, unravel new molecular functions and deorphanize putative targets. Yet, it is well documented that to avoid costly failures in screening projects, ADMET (Absorption, Distribution, Metabolism, Excretion and Toxicity) properties should be considered at an early stage [1-3]. In general, molecules with inadequate properties, even if they do not fail in clinical trials, usually increase the development costs and put significant burden on patients, since, for instance, poorly absorbed drugs have to be given at a higher dose or shifted from oral to intravenous route. A compound collection may be prepared for a chemical biology project or for drug discovery, and in these cases, one may need molecules with a more "lead-like" or "drug-like" profile [3-5]. Experimental ADMET measurements allow to investigate several mechanisms, from crossing physiological barriers, group reactivity to metabolism. Different experimental assays have been developed over the years to try to assess/predict ADMET properties, but in silico computations can also be carried out to rapidly analyze a compound collection or prior to synthesis. In general, these calculations provide valuable information that can then be further investigated experimentally [6].

The fastest method for evaluating the drug-like or lead-like properties of a compound is to apply simple rules that characterize the molecule. Well-known rules are, for instance, the so-called "rule of 5" (RO5) [7]. These rules are a set of property values that were derived from classifying the key physicochemical properties of drug compounds. Drug-like molecules, according to Dr. Lipinski, refers to compounds that have sufficiently acceptable ADME properties and sufficiently acceptable toxicity properties to survive through the completion of human Phase I clinical trial. Yet, the rule of 5 only underlines properties that would make a compound a likely orally active drug in humans, but clearly these rules do not investigate directly metabolism, probe if a molecule is a frequent hitter or if it contains reactive functional groups. Over the years, many additional rules have thus been proposed [8,9] and can be smartly combined with the "rule of 5". Several outstanding commercial packages from ChemAxon, OpenEye or the Chemical Computing Group have been developed and can be used to perform this kind of filtering. To this end, each compound can be associated with a series of descriptors deduced from its 1D/2D/3D structures while some specific substructures and physicochemical properties can also be investigated.

In this article, we describe a new enhanced version of FAF-Drugs, which was originally based on the free chemoinformatics toolkit Frowns [10]. The first FAF-Drugs version could only be called online to filter out libraries using a simple set of 14 structural and physicochemical rules. The new (Additional file 1) FAF-Drugs2 version has been entirely rewritten in Python, does not use Frowns (i.e., the chemistry toolkit is no longer maintained) but imports modules from the OpenBabel toolkit. In its current version, FAF-Drugs2 still provides the basic physicochemical rules computed in the previous version, but it now has many additional features. For instance, it allows selection based upon the number of heavy atoms, search for toxic or undesirable substructures, and permit to flag oral bioavailability using Lipinski's [7], Veber's [8] and Egan's [11] rules. With regard to substructure search, we, for the time being, aim at identifying key toxic functional groups and some toxic and unstable molecules (or groups). We detect 22 "warhead" chelators [12,13], 15 frequent hitters [14], 48 promiscuous inhibitors [15,16] and 116 other key functional groups [17-19]. The package can now be downloaded and users can install it on their workstations and implement new rules as needed.

## Implementation

FAF-Drugs2 consists of a set of object oriented Python modules, some of them importing methods from the OpenBabel toolkit. This is made possible through the OpenBabel Python module Pybel that provides to "FAF-Drugs2" access to the OpenBabel C++ library [20].

There are several reasons why we chose to design the software using Python programmable scripting language and the OpenBabel chemoinformatics toolkit. Python is widely used in the scientific community and as such FAF-Drugs2 was developed to be user friendly for the scientists. Furthermore, Python can easily connect external modules written in other languages, hence using facilities of the OpenBabel toolkit. Actually, OpenBabel is a C++ toolkit [21], designed to read, convert, write and dissect molecular files as well as to compute/predict some useful descriptors such as log*P *values, molecular weight while allowing SMARTS substructure search through implementation of the SMARTS language [22]. Moreover all functionalities can be accessed, imported and integrated in our toolkit by using the Pybel module [20]. We indeed aim at providing a free, collaborative and customizable software that could evolve as knowledge and awareness about ADMET increase.

In order to generate a compound collection with acceptable physicochemical properties, several filtering rules are applied, including the well-known Lipinski's rule-of-five (poor oral absorption if the molecular weight is more than 500, log*P *or octanol/water partition coefficient must not be more than 5, H-bond donors must not be more than 5 and H-bond acceptors must not be more than 10) [7]. The main properties computed by FAF-Drugs2 are: the number of rigid and flexible bonds [8], TPSA (topological Polar Surface Area) value according to Ertl *et al*[23], the number and maximum size of system rings, and the presence of unwanted chemicals or chemical substructures (i.e., carried out using SMARTS searches).

### FAF-Drugs2 modules

**Figure 1:**
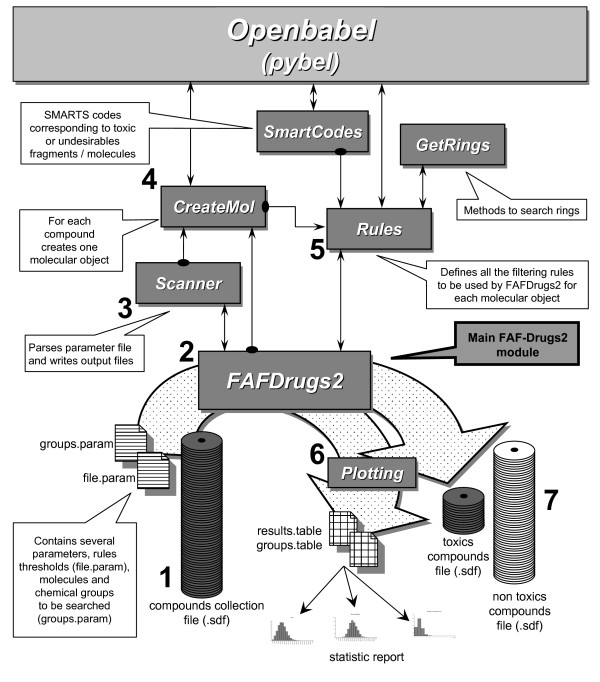
**Schematic diagram of the FAF-Drugs2 package architecture**. FAF-Drugs2 requires as input files a compound collection in SDF format and two parameter files, one containing physicochemical thresholds and the other, the list of substructures that have to be investigated. Several output files are generated, among others, one containing the molecules that do pass the filters and another one, the molecules that do not pass the test. In addition, a table file reporting all the computed values/descriptors for each compound is provided (see text for explanations).

The top-level objects and modules of FAF-Drugs2 are illustrated in Figure 1. Briefly, the architecture of the program is as follow:

#### Scanner

Performs parsing of the parameter files, writing of the output files and removing of duplicate molecules. Furthermore, if the compound libraries contain salts and counterions, this module applies the desalt utility of Pybel.

#### CreateMol

Creates molecular objects ready to be filtered.

#### Rules

Defines all the filtering rules. This module computes the following descriptors:

A) Directly computed or predicted by using OpenBabel abilities:

(1) Molecular weight (part of Lipinski's RO5).

(2) Number of rotatable bonds.

Defined as any single non-ring bond, bounded to non-terminal heavy atoms according to Veber et al [8]. The amide O = C-N bonds are not considered because of their high rotational energy barrier.

(3) Number of rigid bonds.

(4) Number of heavy atoms.

(5) Number of atom with a net charge.

(6) Sum of formal charges.

(7) Number of carbon atoms (c), (8) number of hetero-atoms (het), (9) Value of ratio het/c.

(10) Specific atoms which are undesirable.

(11,12,13,14) SMARTS substructure searching for functional groups and undesirable molecules:

(a) Reactive or undesirable functional groups according to Rishton *et al *and Oprea *et al*. [9,12,13]

(b) Promiscuous inhibitors according to McGovern *et al*. [24] and Seidler *et al*. [16]

(c) Frequent hitters compounds according to Roche *et al*. [14]

(d) Other functional groups according to Sirois *et al*. [17]

(15) log *P *(part of Lipinski's RO5).

For small collections, the log*P *value is computed by OpenBabel according to Wildman *et al*. [20,25]. If the collection is larger than 10,000 molecules, log*P *should be predicted through the X-Score package (freely available through a license agreement after registration, information can be found at ), in this case XlogP values are computed as described in [26].

(16,17) Numbers of Hydrogen bond donors and hydrogen bond acceptors (part of Lipinski's RO5), computed by using SMARTS expression.

B) Other descriptors computed by using in house Python methods.

(18) Topological Polar Surface Area (TPSA) according to the method developed by Ertl *et al*. [23].

(19) Number of rings and (20) maximum size of ring, computed by using in house methods from the *GetRings *module.

(21) Number of violations of Lipinski's RO5 according to Lipinski *et al*. [7].

(22) Veber Rule: defined as TPSA>140Å or number of rotatable bonds>10 [8].

(23) Egan Rule: defined as TPSA > 131.6Å or log *P *> 5.88 [11].

#### SmartsCodes

Contains SMARTS patterns for detection of hydrogen bond donors, hydrogen bond acceptors and all SMARTS patterns for functional groups and undesirable molecules.

##### GetRings

Contains all functions dedicated to identify the smallest ring systems and the maximum size of rings.

#### FAFDrugs2

This is the main module of FAF-Drugs2. It manages molecule through creation of objects, filters the compounds and deals with input and output.

### Data sets and program parameters

Compound libraries must be in a standard SDFile format [27]. Before running FAF-Drugs2, users should edit and check two parameter files named, faf2.param and groups.param. The first one, faf2.param, contains, among others, the input file location, the path to X-Score executable file if needed, and all the physicochemical threshold values. The second one, groups.param, contains filtering rule thresholds for detection of functional groups and for undesirable compounds/groups (e.g., Michael acceptors, nitro or aldehyde).

## Results

**Figure 2:**
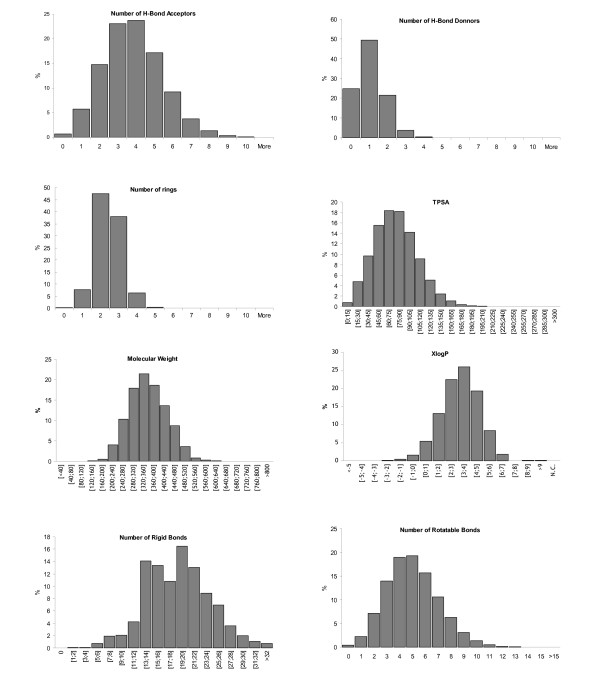
**Analysis of the main physicochemical properties of compound from the ChemBridge EXPRESS-Pick™ Database**. Physicochemical profile of the ChemBridge molecules as computed by FAF-Drugs2.

FAF-Drugs2 is a computer tool (Fig. 1) that helps preparing compound collections. In order to give an example of how to apply this package, we decided to process a large compound collection, namely the June 2008 ChemBridge EXPRESS-Pick™ Database, and provide the key output data obtained by FAF-Drugs2 [28]. This ChemBridge library contains 457,761 compounds. We performed tests on a Linux machine (Dell Precision 690, Bi-Xeon 3 Ghz processors, 2 GB DDRAM, running the CentOS 5 operating system) with the default parameter files. The general properties for this collection (which does not contain any duplicate) as computed by FAF-Drugs2 indicates, among others, that 3,56 % (16315) of the molecules are associated with salts/counterions. Further, figure 2 displays the distribution of the main physico-chemical descriptors for the EXPRESS-Pick™ Database. According to these descriptors, we observed that, 1.98 % (9077) of the molecules have molecular weight over 500, 10,65 % (48769) of the molecules have a XLog*P *value over 5, 0.02 % (85) of the molecules have more than 5 H-Bond donors, 0.04 % (179) of the molecules have more than 10 H-Bond acceptors, 1,74 % (7950) of the molecules have TPSA value over 150, 0.9 % (3978) of the molecules have more than 10 rotatable bonds and finally 0.03% (181) of the molecules contain more than 5 system rings.

**Figure 3:**
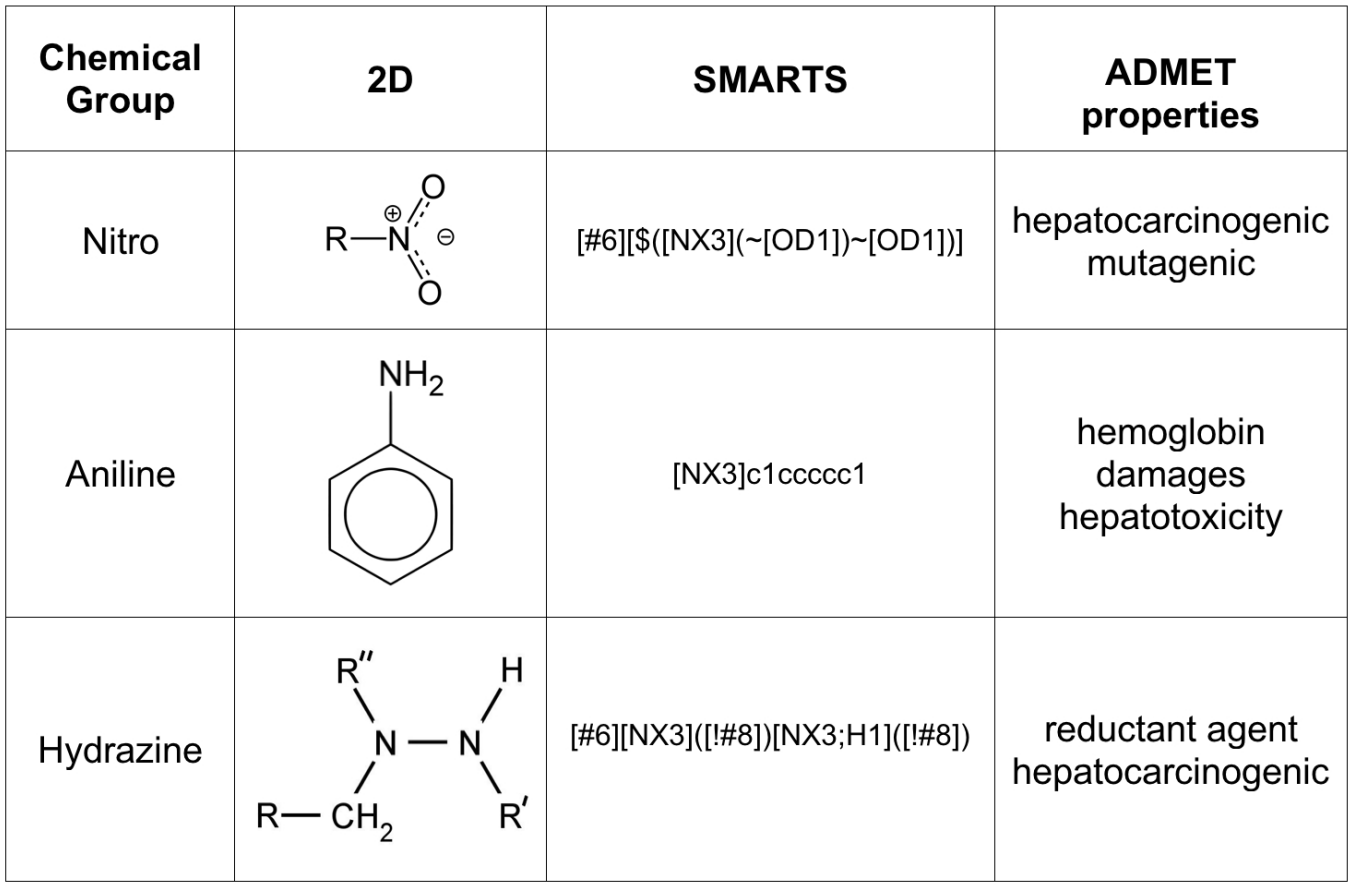
**Examples of undesirable substructures/functional groups assessed by FAF-Drugs2**. Many functional groups/molecules can be problematic for drug discovery projects. Here we illustrate this point with three undesirable groups/compounds searched by FAF-Drugs2 (see Text for details).

Along the same line of reasoning, we illustrate the way FAF-Drugs2 deals with some functional groups (Fig. 3). For example, nitro derivatives have been reported to be hepatocarcinogens [29] and nitroaromatics are reduced to form reactive, nitroanion radical, nitroso intermediate, and N-hydroxy derivative [30]. These reactive metabolites are usually not welcome in drug discovery projects and molecules containing nitroaromatic groups are in general removed from a compound collection or the group will have to be modified later on with another electronwithdrawing group such as trifluoromethyl by chemical synthesis. Yet, it is important to note that some marketed drugs display such a group, like the benzodiazepines, nitrazepam and flunitrazepam and the anti-androgens flutamide and nilutamide [31], among others. When investigating the ChemBridge collection for the presence of nitro groups, we found 48801 molecules (10.8%) containing at least one occurrence of this chemical function.

Also, although a few hydrazinic drugs are used, this group has been regularly associated with hepatoxicity and carcinogenesis [32]. We found 5996 molecules (1.31%) that contain at least one hydrazine group.

Furthermore, some simple compounds can have dramatic effects on Human, for instance some molecules are well known to be carcinogens [33]. For example, aniline or arylamine, a simple aromatic amine, is toxic by inhalation and absorption. This group is regularly pointed out as able to generate toxic metabolites. In addition, some polymorphisms in the N-acetyltransferase 1 (NAT1, one of the major hepatic phase II enzymes) are associated with rapid/slow acetylation phenotypes with altered formation of acetylamino metabolites and increased susceptibility to cancers [34]. However, the arylamine moieties are part of drugs like tacrine and sulfamethoxazole where, in several cases, the amino group has been linked with the hepatotoxity of these drugs [35,36]. We found 232447 (50.8%) compounds in the ChemBridge collection containing one aniline group.

Also, the number of occurrences of some chemical groups could impede the development of molecules that have to be given through oral route. For instance, it is often considered that the number of occurrence of OH groups should not be over 4 or 5 or 6 [37]. It is expected that such polyhydroxylated molecules display poor bioavailability, which is probably a consequence of their rapid metabolic transformation in the liver and gastrointestinal tract [38]. Indeed, we ran FAF-Drugs2 on 4567 drug compounds available at DrugBank [39,40] (small molecule drugs, July 2008, 171 duplicates seem to be present in this library) and found that 501 molecules have more than 3 OH groups and only 144 molecules have more than 5 OH groups. We also carried out this investigation over the ChemBridge collection and we found that 63 compounds (0.01%) possess more than 3 hydroxyl groups.

## Discussion

Drug discovery is an interdisciplinary, expensive and time-consuming process and chemical biology projects share a lot of the difficulties seen in drug discovery programmes. Advances in computational techniques and hardware solutions have enabled in silico methods, and in particular virtual screening, to speed-up modern hit identification and optimization. In most cases, it seems beneficial to run in silico ADMET prediction prior to or after initial screening experiments. Numerous observations over drug compounds have been made over the years leading to a set of rules that can be applied to a compound library or list of molecules, assuming one has appropriate computer methods to parse and dissect each molecule. The structure/atomic composition determine some of the compound's properties. Interaction of the structural properties of a molecule with its physical environment cause physicochemical properties that can be measured experimentally or estimated in silico. Interactions of the structural properties of compounds with molecules/enzymes cause biochemical properties such as metabolism. Ultimately, when the physicochemical and the biochemical properties interact with a living system they can cause toxicity [41]. From these remarks, it is obvious that simple rules cannot fully estimate pharmacokinetics (e.g., half life, clearance...) nor toxicity, yet, investigations of fundamental physicochemical and/or simple biochemical properties together with structure and substructure analyses provide valuable information and allows for the in silico filtering of a compound collection.

In the present study we present an ADMET filtering package, called FAF-Drugs2, written in Python, which can help preparing a compound collection. The library is loaded into the engine as a SDF file and the program, together with two user-defined parameter files, outputs a "non-toxic file" and a "toxic file" that contain respectively, compounds that do pass the filters and compounds that do not satisfy the rules. In addition, the program writes, in a table format, a full report called "table.results", a report of the substructure search called "groups.table" and a file called "summary.txt" summarizing information about each compound. Finally, the users can have the program plot some of the main physicochemical properties to facilitate graphical analysis of the library. The FAF-Drugs2 package can easily be tuned according to a given project. It has been tested successfully on several libraries (not shown) including the ChemBridge compound collection.

## Conclusion

We have developed the FAF-Drugs2 package to facilitate compound collection preparation. Users can select to apply various filtering thresholds and add rules as needed. This application is suitable for conducting filtering of large compound collections and run on Linux platforms. The FAF-Drugs2 package is freely available.

## Availability and requirements

• **Project name: **FAF-Drugs2

• **Project home page: **

• **Operating system(s): **Linux

• **Programming language: **Python

• **Other requirements: **Python 2.5.1 or higher, OpenBabel 2.1.1, GnuPlot 4.2.3 (optional), X-Score (optional).

• **License: **GNU GPL

• **Any restrictions to use by non-academics: **None

## Authors' contributions

DL wrote the FAF-Drugs2 Python package, tested the program and drafted the first version of the manuscript. OS and MAM optimized some methods and tested the program. HG and BOV investigated some chemical groups. BOV initiated the project and highlighted the importance of developing a free ADMET toolkit. All authors took an active part in manuscript writing. All authors read and approved the final manuscript.

## Supplementary Material

Additional file 1 This file contains the FAF-Drugs2 package and user manual. Click here for file
