# Supplementary material for: FAF-Drugs2: Free ADME/tox filtering tool to assist drug discovery and chemical biology projects
Source: BMC Bioinformatics. 2008 Sep 24;9:396. doi: 10.1186/1471-2105-9-396 (PMC2561050; doi:10.1186/1471-2105-9-396)
Supplement: Additional file 1 — This file contains the FAF-Drugs2 package and user manual. [file 1471-2105-9-396-S1.gz › FAFDrugs2/docs/UserGuide_FAF-Drugs2.html]

FAFDrugs2


|  |  |
| --- | --- |
| |  | | --- | |  | |

  

FAF-Drugs2

User
Guide

 

 

 

 

INSERM – MT*i*

Paris Diderot University

35, rue Hélène BRION

75205 Paris Cedex 13

France

 

### CONTENTS

 

What is FAF-Drugs2?

Availability and requirements

How to install FAF-Drugs2?

How to run FAF-Drugs2?

Example

References

### What is FAF-Drugs2?

# FAF-Drugs2 (Free ADME-Tox Filtering version 2) is a program for filtering large compound libraries prior to in silico screening experiments or related modeling studies. The main goal is the computational prediction of some ADME-Tox properties (Adsorption, Distribution, Metabolism, Excretion and Toxicity)[1]. It is a free toolkit to assist in silico screening experiments as well as experimental screening as it helps to select compounds for in silico/in vitro/in cellulo assays. The package rests on a combination of Python modules, PyADME, Scanner, CreateMol, Rules, GetRings and SmartsCodes, creating an interface between the user and the OpenBabel toolkit [2], via the Pybel module which provides access to the OpenBabel C++ library [3]. FAF-Drugs2 has been developed in the INSERM–University Paris Diderot lab called MTi (see http://www.vls3d.com and also (in construction): http://www.mti.univ-paris-diderot.fr/). The package FAF-Drugs2 is written in Python [4] and is available for Linux system under the GNU General Public License. We are working on a Mac OS X version as well.

Please, cite:

David Lagorce, Olivier Sperandio, Hervé Galons, Maria A
Miteva, Bruno O Villoutreix.

**FAF-Drugs2 : a free
ADME/tox filtering tool to assist drug discovery and chemical biology projects**

You may direct questions related to this package to the
corresponding author:

**Bruno O Villoutreix**

INSERM –
Paris Diderot University – MT*i*

```
35, rue Hélène BRION
```

```
75205 Paris Cedex 13
```

*E-mail :* bruno.villoutreix@univ-paris-diderot.fr

### Availability and requirements

 

Project
home page: http://www.vls3d.com/FAF2/FAFDrugs2.tar.gz

Operating
system: Linux

Programming
language: Python

Other
requirements:

- Python 2.5.1 (at least) freely available at:

http://www.python.org/download/

 

- OpenBabel release 2.1.1 included in one extra directory
in the FAF-Drugs2

package and freely available at:

http://sourceforge.net/project/showfiles.php?group\_id=40728

 

- Gnuplot 4.2.3 [5] (also provided here, drawing
tool) freely available at:

http://www.gnuplot.info/download.html

 

-
X-Score [6] (to computer for instance log
P), freely available under license agreement at (we do not provide it, you need
to contact the authors, one default method to computer log P is however
implemented in the present distribution, yet it would be important to have
X-Score):

http://sw16.im.med.umich.edu/software/xtool/manual/download.html

 

License:
GPL

Any
restrictions to use by non-academics: None

 

### How to install FAF-Drugs2?

 

**Steps 1 to 4 are** **REQUIRED.**

In your home directory, we assume you do not have OpenBabel
and Python packages installed.

**Step 1: In
your home directory, uncompress the FAF-Drugs2 package in a Linux shell
(**tar -xvzf FAFDrugs2.tar.gz).

It
creates one directory named “/FAF-Drugs2” containing the subdirectories:

~/bin                            Contains Python executables files
and modules. ~/extras                        Contains
the OpenBabel 2.1.1 package. This is the

directory where
you can install OpenBabel and

Python
programming language. As well, here you

can deposit
and install X-Score and GnuPlot

package. In this case, please see
sections 5-6.

~/parameters\_files        Contains soft and hard generics faf2.param
and

groups.param
that you can customize as you want.

Note that they
must be placed in your working and

modified as
needed.

~/example                    Contains a
small generic dataset for running FAF-Drugs2.

~/doc                           Contains the UserGuide, X-Score
installing

procedure and
a standard FAF-Drugs2 installing

procedure.

IMPORTANT:

Set the executable path for FAF-Drugs2
in your ".bash\_profile" or ".cshrc".

For instance, in my ".bash\_profile"
file I have:

*PATH=$PATH:$HOME/bin:/home/david/FAFDrugs2/bin:*

For ".cshrc", *set path = ($path ~/FAFDrugs2/bin)*

 

**Step 2: After
downloading Python2.5.2.tar.gz, in the extra directory or in your home
directory install the Python programming language package in a Linux
shell** **as follows:**

\*   
tar -xvzf Python-2.5.2.tgz

\*   
cd Python-2.5.2

\*   
./configure

\*   
make

Under root permissions

\*   
make install

Exit from root

 

**Step 3: Go
to the “extras” directory and install the OpenBabel package in a
Linux shell** **as follows:**

\*   
tar -xvzf openbabel.2.1.1.tar.gz

\*   
cd openbabel.2.1.1

\*   
./configure

\*   
make

Under root permissions

\*    make install

Exit from root

 

**Installing
Pybel module is needed to bind Python with OpenBabel :**

\*   
cd scripts/python

\*   
python setup.py build

Under root permissions

\*   
python setup.py install

Exit from root

 

WARNING:

If you are running X86\_64bits architecture
machine you must edit the file
/usr/local/lib/python2.5/site-packages/openbabel.py and replace the line
"import dl" with "import DLFCN as dl"

 

**Step 4: Set
the PATH environment variables** **as follow:**

Edit your ".bashrc" (or ".cshrc") and add these
lines (example for bash shell, please check the internet for additional
explanations or for C shell equivalent):

LD\_LIBRARY\_PATH=/usr/local/lib

export
LD\_LIBRARY\_PATH

 

for ".cshrc" :    setenv LD\_LIBRARY\_PATH /usr/local/lib

Source your ".bashrc" (or ".cshrc")

 

**OPTIONAL (but
important):**

**Step
5: If your compound library exceeds 10.000 molecules, you will need to
install the X-Score package** **as follow
(this is to computer log P values for a large collection, the default tool
implemented in OpenBabel has a bug, this will be solved soon, in any case,
X-score is a must to have):**

After registration, you can
download the software, **put it in the
“extras” directory and install it as follow**. Note that you need to
place your license agreement file in your home directory.

\*    tar -xvzf xscore.tar.gz

\*    cd xscore\_v1.2.1/c++/

\*    make

\*    cp xscore ../bin/

 

You need to set the X-Score
environment variables.

Add these lines to your ".bash\_profile":

            1)

export XTOOLS\_HOME=$XTOOLS\_HOME:***"FAFDrugs2 path
install…"***

***for
instance on my machine I have:***

***export
XTOOLS\_HOME=$XTOOLS\_HOME:/home/david/FAFDrugs2/extras/xscore\_v1.2.1***

export XTOOLS\_PARAMETERS=$XTOOLS\_HOME/parameters

export XTOOLS\_BIN=$XTOOLS\_HOME/bin

2)

Add the
executable path for X-Score in your ".bash\_profile", for instance:

***PATH=$PATH:$HOME/bin:/home/david/FAFDrugs2/******extras/xscore\_v1.2.1/bin:***

 

or to your ".cshrc":

1)

setenv XTOOL\_HOME  
the\_installation\_directory\_of\_X-Score

***for instance
XTOOL\_HOME  /home/david/FAFDrugs2/extras/xscore\_v1.2.1***

setenv XTOOL\_PARAMETER 
$XTOOL\_HOME/parameter

setenv XTOOL\_BIN 
$XTOOL\_HOME/bin

set path = ($path  $XTOOL\_BIN)

2)

*set path = ($path ~/FAFDrugs2/extra/**xscore\_v1.2.1/bin)*

 

Source your ".bash\_profile" (or ".cshrc")

 

**test if X-Score can be executed in a shell with the command “xscore”.**

 

NOTE: if you are going to use X-Score toolkit, please check
that in the file <faf2.param> you have switched the xscore flag to
"on"

 

**Step 6: After
downloading GnuPlot 4.2.3, put it in the “extras” directory and
install it as follow:**

\*   
tar -xvzf gnuplot-4.2.3.tar.gz

\*   
cd gnuplot-4.2.3

\*   
./configure

\*   
make

Under root permissions

\*   
make install

Exit from root

 

 

**test if Gnuplot can be executed in a shell with the command “gnuplot”**

 

If is not possible, add the
absolute path of the gnuplot executable file in your searching PATH (for
instance, in ".bash\_profile" add the gnuplot executable path to
the line: PATH=$PATH:$HOME/bin:)

Ø     
source  .bash\_profile

 

**NOTE:**

If you are going to use the GnuPlot toolkit, check that in
the file <faf2.param> (see parameters files section) you have switched
the gnuplot flag to "on".

  

How to run FAF-Drugs2 ?

 

You can run the processes
anywhere you want if you have added the absolute path of the FAF-Drugs2 bin
in your searching PATH.

 

The directory where you want to
use FAF-Drugs2 must contain:

-        
compounds collection (.sdf file required)

-        
faf2.param

-        
groups.param

 

**In the parameters\_files directory you will find enclosed generics parameters
files with soft and/or hard thresholds. For convenience in this userguide, the
faf2\_soft.param and faf2\_hard.param are called faf2.param and the
groups\_soft.param and groups\_hard.param are called groups.param**

 

**Input File**

 

*WARNING:*

*Sometimes, some input files must be processed by the internal
“dos2unix” tool to be acceptable. This has to do with the way lines are ended.*

 

Compounds collection must be a
valid SD File [7]. It means header block of
each molecule must be on 3 lines (maximum), the first one must contain the
molecule name or the ID tag **in a one
continuous name.**

If no name is specified, you must
perform this adjustment.

If you want you can use the extra
tool called "SDF\_ID\_Formatter.py", **enclosed in the "extras" directory of the FAF-Drugs2
package**. It will automatically generates a new file named "<*your\_inputfile*>\_formatted.sdf"

Before using it, you need to get
the ID field (grey mark):

- example 1:

            >  <ID>

            10156

 

- example 2:

            >  <ID> (10156)

            10156

 

command line:

Ø     
python SDFFormatter.py "<*ID field>*" <.sdf input file
to process>

 

 

For supplementary support please
contact David Lagorce at:

david.lagorce@univ-paris-diderot.fr

 

**Parameters files**

**\*   
faf2.param**

**REQUIRED:**

Some fields must be filled:

Ø     
X-Score

If you treat a compound collection exceeding 10000 molecules, you
should use the X-Score toolkit to prevent a memory leak and OpenBabel program
abnormal termination. This is a known problem due to the log*P* prediction method implemented in the
present version of the OpenBabel toolkit. This should be fixed in the next
stable version of OpenBabel. In this case, set the flag to "on".

 

xscore =
"on" or "off "

xscore\_path = <the executable file path of xscore >

*for instance on my machine:*

*/home/david/FAFDrugs2/extras/xscore\_v1.2.1/bin*

 

Ø     
GnuPlot

If you want to
get some histograms related to some descriptor distributions in your compound
collection, you need to set the plotting flag to "on"

 

Ø     
File to process

You must give the name of the
compound collection

           

Ø     
Group param file

You must indicate the name of the
chemical groups you want to test in the param file. As it is, we are providing
a long list of chemicals, as such you can use the default parameters to start
with.

 

You can also tune the thresholds of many parameters in the
faf2.param according to your project and goals.

**\*  
 groups.param**

 

You can customize this file to
tune the substructure search. Adding one "#" before a substructure
will preclude it from being searched/tested. But, you can also choose the number
of corresponding substructure allowed in each compound tested:

            Syntaxe
:          (#) 0                 Imine

                                 *yes/no           
substructure*

*number*

NOTE:

You can customize the
SmartsCode.py module by adding your own SMARTS pattern in a similar manner.
This implies that you must add the corresponding substructure name in the
groups.param file the same way it is mentioned above.

  

 

|  |
| --- |
| NOTE:  FAF-Drugs2 toolkit computes Lipinski’s, Veber’s and Egan’s rules violations, such that users must specify coherent rules’ thresholds. For instance, Veber’s rule states that TPSA must be lower than 140 or number of rotatable bonds must be lower than 10, so if the user specifies a value of 150 for TPSA **and** a value of 20 rotatable bonds, the program will bypass this evaluation. |

 

 

 

**Running FAF-Drugs2**

 

To run FAF-Drugs2 you must use in
a terminal window the command:

> FAFDrugs2.py
faf2.param

Results are automatically written
in a new directory, the output directory is named:

"FAF-Drugs2\_OUTPUT*\_Month\_Day\_Year\_Hour*"

  

Example

 

You
will find enclosed in the "example" directory all you need to test
this package:

Ø     
1000mol.sdf (the input file)

Ø     
faf2.param (generic parameter file)

*warning**: to perform one test, you must adjust in this file your
xscore path.*

Ø     
groups.param (generic groups parameter file)

Once
the process is terminated, the output directory is automatically created and
named. FAF-Drugs2\_OUTPUT\_*Month\_Day\_Year\_Hour*
contains:

 

Ø     
toxic.sdf (contains toxic compounds or compounds
that do not pass the filters)

Ø     
non\_toxic.sdf
(contains non-toxic compounds)

Ø     
summary.txt (contains all compounds in SMILE format
with ADMET status)

Ø     
results.table (contains the whole ADMET results
for each compound)

Ø     
groups.table (contains the whole substructure
hits for each compound)

Ø     
compounds\_collection\_without\_salts.sdf
(compounds collection with no salts)

Ø     
different gnuplot histograms (.png) if you need
them.

Ø     
faf2.param\_used (file param you used for this
filtering)

Ø     
groups.param\_used (groups.param you used for
this filtering)

*optional:*

Ø     
*compound
collection without duplicates.*

Ø     
*compound
collection without duplicates and without salts.*

 

 

 

 

 

 

 

 

 

 

 

 

 

 

 

 

 

 

 

 

  

References

 

1.         Lagorce D, Sperandio O,
Galons H, Miteva M, Villoutreix B: **FAF-Drugs2:
free ADME/tox filtering tool to assist drug discovery and chemical biology
projects**. 2008.

2.         OpenBabel:
**Open Babel 2.1.1 - http://openbabel.org/wiki/Main\_Page**.
In*.*, Open Babel 2.1.1 edn; 2008.

3.         O'Boyle
NM, Morley C, Hutchison GR: **Pybel: a
Python wrapper for the OpenBabel cheminformatics toolkit**. *Chem Cent J* 2008, **2**:5.

4.         Python:
**Python Programming Language - http://www.python.org/**.

5.         Gnuplot:
**Gnuplot - http://www.gnuplot.info/**.

6.         Wang
R: **Calculating partition coefficient by
atom-additive method**. *Perspectives in
Drug Discovery and Design* 2000, **19**:47–66.

7.         **SD File format - http://www.mdl.com/downloads/public/ctfile/ctfile.pdf**.
In*.*: MDL - Symix; 2007.
