# Supplementary material for: FAF-Drugs2: Free ADME/tox filtering tool to assist drug discovery and chemical biology projects
Source: BMC Bioinformatics. 2008 Sep 24;9:396. doi: 10.1186/1471-2105-9-396 (PMC2561050; doi:10.1186/1471-2105-9-396)
Supplement: Additional file 1 — This file contains the FAF-Drugs2 package and user manual. [file 1471-2105-9-396-S1.gz › FAFDrugs2/docs/UserGuide_FAF-Drugs2.pdf]

Molécules Thérapeutiques *in silico*

FAF-Drugs2
User Guide

INSERM – MT*i*
Paris Diderot University
35, rue Hélène BRION
75205 Paris Cedex 13
France

CONTENTS

What is FAF-Drugs2?
Availability and requirements
How to install FAF-Drugs2?
How to run FAF-Drugs2?
Example
References

What is FAF-Drugs2?

FAF-Drugs2 (Free ADME-Tox Filtering version 2) is a program for filtering large compound libraries prior to in silico screening experiments or related modeling studies. The main goal is the computational prediction of some ADME-Tox properties (Adsorption, Distribution, Metabolism, Excretion and Toxicity)[1]. It is a free toolkit to assist in silico screening experiments as well as experimental screening as it helps to select compounds for in silico/in vitro/in cellulo assays. The package rests on a combination of Python modules, PyADME, Scanner, CreateMol, Rules, GetRings and SmartsCodes, creating an interface between the user and the OpenBabel toolkit [2], via the Pybel module which provides access to the OpenBabel C++ library [3]. FAF-Drugs2 has been developed in the INSERM–University Paris Diderot lab called MTi (see http://www.vls3d.com and also (in construction): http://www.mti.univ-paris-diderot.fr/). The package FAF-Drugs2 is written in Python [4] and is available for Linux system under the GNU General Public License. We are working on a Mac OS X version as well.

Please, cite:
David Lagorce, Olivier Sperandio, Hervé Galons, Maria A Miteva, Bruno O Villoutreix.
FAF-Drugs2 : a free ADME/tox filtering tool to assist drug discovery and chemical biology projects

You may direct questions related to this package to the corresponding author:
Bruno O Villoutreix
INSERM – Paris Diderot University – MT*i*
35, rue Hélène BRION
75205 Paris Cedex 13
E-mail : bruno.villoutreix@univ-paris-diderot.fr

Availability and requirements

Project home page: http://www.vls3d.com/FAF2/FAFDrugs2.tar.gz
Operating system: Linux
Programming language: Python
Other requirements:

- Python 2.5.1 (at least) freely available at:
  http://www.python.org/download/

- OpenBabel release 2.1.1 included in one extra directory in the FAF-Drugs2 package and freely available at:
  http://sourceforge.net/project/showfiles.php?group_id=40728

- Gnuplot 4.2.3 [5] (also provided here, drawing tool) freely available at:
  http://www.gnuplot.info/download.html

- X-Score [6] (to computer for instance log P), freely available under license agreement at (we do not provide it, you need to contact the authors, one default method to computer log P is however implemented in the present distribution, yet it would be important to have X-Score):
  http://sw16.im.med.umich.edu/software/xtool/manual/download.html

License: GPL
Any restrictions to use by non-academics: None

How to install FAF-Drugs2?

Steps 1 to 4 are <u>REQUIRED.</u>
In your home directory, we assume you do not have OpenBabel and Python packages installed.

Step 1: In your home directory, uncompress the FAF-Drugs2 package in a Linux shell (tar -xvzf FAFDrugs2.tar.gz).
It creates one directory named "/FAF-Drugs2" containing the subdirectories:

~/bin	Contains Python executables files and modules.
~/extras	Contains the OpenBabel 2.1.1 package. This is the directory where you can install OpenBabel and Python programming language. As well, here you can deposit and install X-Score and GnuPlot package. In this case, please see sections 5-6.
~/parameters_files	Contains soft and hard generics faf2.param and groups.param that you can customize as you want. Note that they must be placed in your working and modified as needed.

~/example	Contains a small generic dataset for running FAF-Drugs2.
~/doc	Contains the UserGuide, X-Score installing procedure and a standard FAF-Drugs2 installing procedure.

IMPORTANT:
Set the executable path for FAF-Drugs2 in your ".bash_profile" or ".cshrc".
For instance, in my ".bash_profile" file I have:
PATH=$PATH:$HOME/bin:/home/david/FAFDrugs2/bin:
For ".cshrc", *set path = ($path ~/FAFDrugs2/bin)*

Step 2: After downloading Python2.5.2.tar.gz, in the extra directory or in your home directory install the Python programming language package in a Linux shell as follows:

* tar -xvzf Python-2.5.2.tgz
* cd Python-2.5.2
* ./configure
* make

Under root permissions

* make install

Exit from root

Step 3: Go to the “extras” directory and install the OpenBabel package in a Linux shell as follows:

* tar -xvzf openbabel.2.1.1.tar.gz
* cd openbabel.2.1.1
* ./configure
* make

Under root permissions

* make install

Exit from root

Installing Pybel module is needed to bind Python with OpenBabel :

* cd scripts/python
* python setup.py build

Under root permissions

* python setup.py install

Exit from root

WARNING:
If you are running X86_64bits architecture machine you must edit the file /usr/local/lib/python2.5/site-packages/openbabel.py and replace the line "import dl" with "import DLFCN as dl"

Step 4: Set the PATH environment variables as follow:

Edit your ".bashrc" (or ".cshrc") and add these lines (example for bash shell, please check the internet for additional explanations or for C shell equivalent):

LD_LIBRARY_PATH=/usr/local/lib
export LD_LIBRARY_PATH

for ".cshrc" :   setenv LD_LIBRARY_PATH /usr/local/lib
Source your ".bashrc" (or ".cshrc")

OPTIONAL (but important):
Step 5: If your compound library exceeds 10.000 molecules, you will need to install the X-Score package as follow (this is to computer log P values for a large collection, the default tool implemented in OpenBabel has a bug, this will be solved soon, in any case, X-score is a must to have):

After registration, you can download the software, **put it in the "extras" directory and install it as follow**. Note that you need to place your license agreement file in your home directory.

* tar -xvzf xscore.tar.gz
* cd xscore_v1.2.1/c++/
* make
* cp xscore ../bin/

You need to set the X-Score environment variables.
Add these lines to your ".bash_profile":
1)
export XTOOLS_HOME=$XTOOLS_HOME:***"FAFDrugs2 path install…"***
for instance on my machine I have:
export XTOOLS_HOME=$XTOOLS_HOME:/home/david/FAFDrugs2/extras/xscore_v1.2.1
export XTOOLS_PARAMETERS=$XTOOLS_HOME/parameters
export XTOOLS_BIN=$XTOOLS_HOME/bin
2)
Add the executable path for X-Score in your ".bash_profile", for instance:
PATH=$PATH:$HOME/bin:/home/david/FAFDrugs2/extras/xscore_v1.2.1/bin:

or to your ".cshrc":
1)
setenv XTOOL_HOME   the_installation_directory_of_X-Score
for instance XTOOL_HOME /home/david/FAFDrugs2/extras/xscore_v1.2.1
setenv XTOOL_PARAMETER $XTOOL_HOME/parameter
setenv XTOOL_BIN $XTOOL_HOME/bin
set path = ($path $XTOOL_BIN)
2)
set path = ($path ~/FAFDrugs2/extra/xscore_v1.2.1/bin)

Source your ".bash_profile" (or ".cshrc")

test if X-Score can be executed in a shell with the command "xscore".

NOTE: if you are going to use X-Score toolkit, please check that in the file <faf2.param> you have switched the xscore flag to "on"

Step 6: After downloading GnuPlot 4.2.3, put it in the “extras” directory and install it as follow:

- tar -xvzf gnuplot-4.2.3.tar.gz
- cd gnuplot-4.2.3
- ./configure
- make

Under root permissions

- make install

Exit from root

test if Gnuplot can be executed in a shell with the command “gnuplot”

If is not possible, add the absolute path of the gnuplot executable file in your searching PATH (for instance, in ".bash_profile" add the gnuplot executable path to the line: PATH=$PATH:$HOME/bin:)

- ➢ source .bash_profile

NOTE:
If you are going to use the GnuPlot toolkit, check that in the file <faf2.param> (see parameters files section) you have switched the gnuplot flag to "on".

How to run FAF-Drugs2 ?

You can run the processes anywhere you want if you have added the absolute path of the FAF-Drugs2 bin in your searching PATH.

The directory where you want to use FAF-Drugs2 must contain:

- compounds collection (.sdf file required)
- faf2.param
- groups.param

In the parameters_files directory you will find enclosed generics parameters files with soft and/or hard thresholds. For convenience in this userguide, the faf2_soft.param and faf2_hard.param are called faf2.param and the groups_soft.param and groups_hard.param are called groups.param

Input File

WARNING:
Sometimes, some input files must be processed by the internal "dos2unix" tool to be acceptable. This has to do with the way lines are ended.

Compounds collection must be a valid SD File [7]. It means header block of each molecule must be on 3 lines (maximum), the first one must contain the molecule name or the ID tag **in a one continuous name.**
If no name is specified, you must perform this adjustment.
If you want you can use the extra tool called "SDF_ID_Formatter.py", **enclosed in the "extras" directory of the FAF-Drugs2 package**. It will automatically generates a new file named "<*your_inputfile*>_formatted.sdf"
Before using it, you need to get the ID field (grey mark):
- example 1:

    > <ID>
    10156

- example 2:

    > <ID> (10156)
    10156

command line:

- python SDFFormatter.py "<*ID field*>" <.sdf input file to process>

For supplementary support please contact David Lagorce at:
david.lagorce@univ-paris-diderot.fr

Parameters files

* faf2.param

REQUIRED:
Some fields must be filled:

- X-Score
  If you treat a compound collection exceeding 10000 molecules, you should use the X-Score toolkit to prevent a memory leak and OpenBabel program abnormal termination. This is a known problem due to the log*P* prediction method implemented in the present version of the OpenBabel toolkit. This should be fixed in the next stable version of OpenBabel. In this case, set the flag to "on".

  xscore = "on" or "off "
  xscore_path = <the executable file path of xscore >
  for instance on my machine:
  /home/david/FAFDrugs2/extras/xscore_v1.2.1/bin

- GnuPlot
  If you want to get some histograms related to some descriptor distributions in your compound collection, you need to set the plotting flag to "on"

- File to process
  You must give the name of the compound collection

- Group param file
  You must indicate the name of the chemical groups you want to test in the param file. As it is, we are providing a long list of chemicals, as such you can use the default parameters to start with.

You can also tune the thresholds of many parameters in the faf2.param according to your project and goals.

* groups.param

You can customize this file to tune the substructure search. Adding one "#" before a substructure will preclude it from being searched/tested. But, you can also choose the number of corresponding substructure allowed in each compound tested:

Syntaxe :	(#) 0	Imine
	yes/no *number*	*substructure*

NOTE:
You can customize the SmartsCode.py module by adding your own SMARTS pattern in a similar manner. This implies that you must add the corresponding substructure name in the groups.param file the same way it is mentioned above.

NOTE:

FAF-Drugs2 toolkit computes Lipinski's, Veber's and Egan's rules violations, such that users must specify coherent rules' thresholds. For instance, Veber's rule states that TPSA must be lower than 140 or number of rotatable bonds must be lower than 10, so if the user specifies a value of 150 for TPSA **and** a value of 20 rotatable bonds, the program will bypass this evaluation.

Running FAF-Drugs2

To run FAF-Drugs2 you must use in a terminal window the command:

```
> FAFDrugs2.py faf2.param
```

Results are automatically written in a new directory, the output directory is named:

"FAF-Drugs2_OUTPUT_*Month_Day_Year_Hour*"

Example

You will find enclosed in the "example" directory all you need to test this package:

- 1000mol.sdf (the input file)
- faf2.param (generic parameter file)
  warning: to perform one test, you must adjust in this file your xscore path.
- groups.param (generic groups parameter file)

Once the process is terminated, the output directory is automatically created and named.
FAF-Drugs2_OUTPUT_*Month_Day_Year_Hour* contains:

- toxic.sdf (contains toxic compounds or compounds that do not pass the filters)
- non_toxic.sdf (contains non-toxic compounds)
- summary.txt (contains all compounds in SMILE format with ADMET status)
- results.table (contains the whole ADMET results for each compound)
- groups.table (contains the whole substructure hits for each compound)
- compounds_collection_without_salts.sdf (compounds collection with no salts)
- different gnuplot histograms (.png) if you need them.
- faf2.param_used (file param you used for this filtering)
- groups.param_used (groups.param you used for this filtering)

optional:

- *compound collection without duplicates.*
- *compound collection without duplicates and without salts.*

References

1. Lagorce D, Sperandio O, Galons H, Miteva M, Villoutreix B: **FAF-Drugs2: free ADME/tox filtering tool to assist drug discovery and chemical biology projects**. 2008.
2. OpenBabel: **Open Babel 2.1.1 - http://openbabel.org/wiki/Main_Page**. In., Open Babel 2.1.1 edn; 2008.
3. O'Boyle NM, Morley C, Hutchison GR: **Pybel: a Python wrapper for the OpenBabel cheminformatics toolkit**. *Chem Cent J* 2008, **2**:5.
4. Python: **Python Programming Language - http://www.python.org/**.
5. Gnuplot: **Gnuplot - http://www.gnuplot.info/**.
6. Wang R: **Calculating partition coefficient by atom-additive method**. *Perspectives in Drug Discovery and Design* 2000, **19**:47–66.
7. **SD File format - http://www.mdl.com/downloads/public/ctfile/ctfile.pdf**. In.: MDL - Symix; 2007.
