# Supplementary figures and images for: FAF-Drugs2: Free ADME/tox filtering tool to assist drug discovery and chemical biology projects
Source: BMC Bioinformatics. 2008 Sep 24;9:396. doi: 10.1186/1471-2105-9-396 (PMC2561050; doi:10.1186/1471-2105-9-396)

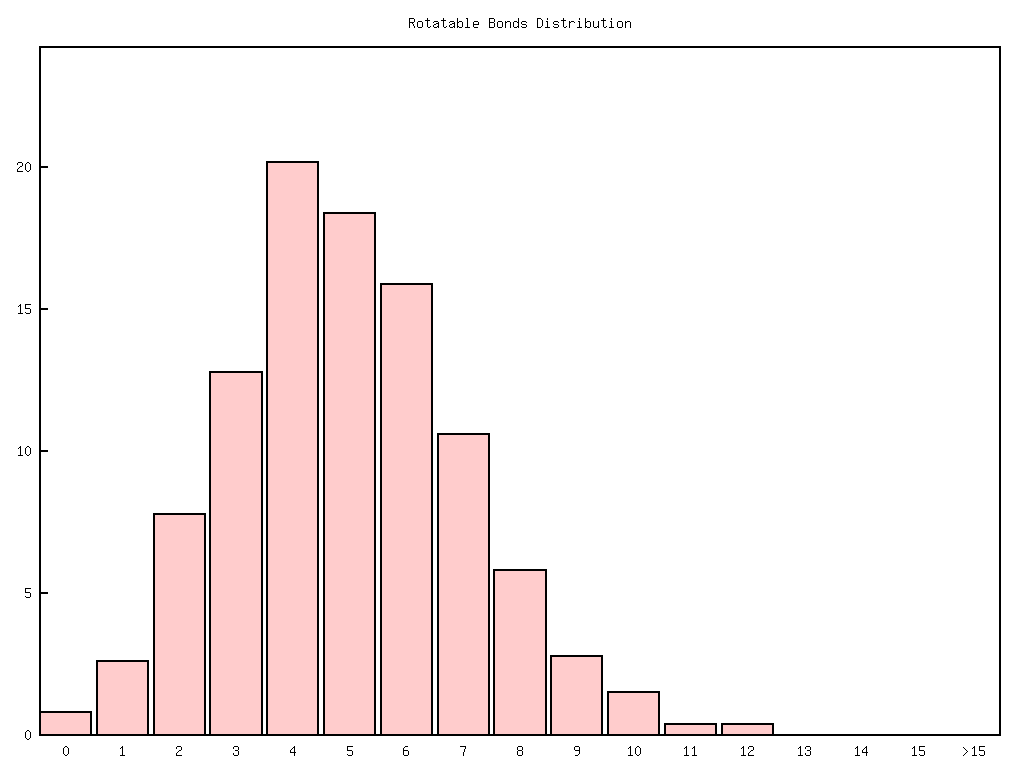

Supplement: Additional file 1 — This file contains the FAF-Drugs2 package and user manual. [file 1471-2105-9-396-S1.gz › FAFDrugs2/example/FAFDrugs2_OUTPUT_Jul_16_2008_17h57m34/RotatableBondsDistribution.png]

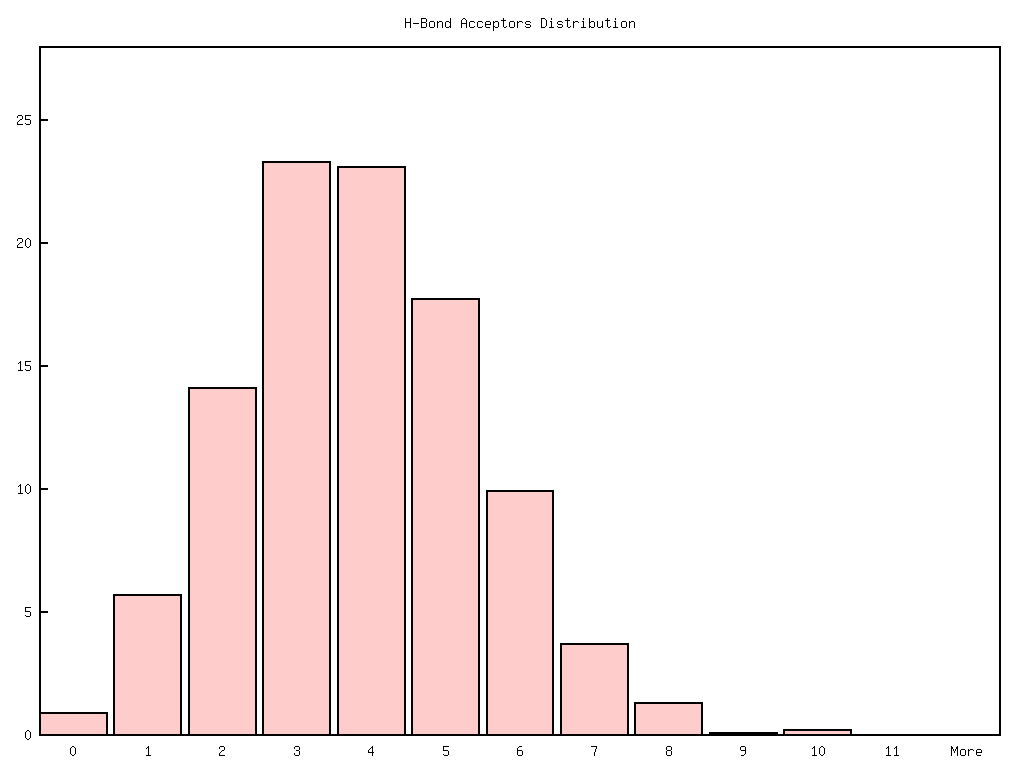

Supplement: Additional file 1 — This file contains the FAF-Drugs2 package and user manual. [file 1471-2105-9-396-S1.gz › FAFDrugs2/example/FAFDrugs2_OUTPUT_Jul_16_2008_17h57m34/HBondAcceptorsDistribution.png]

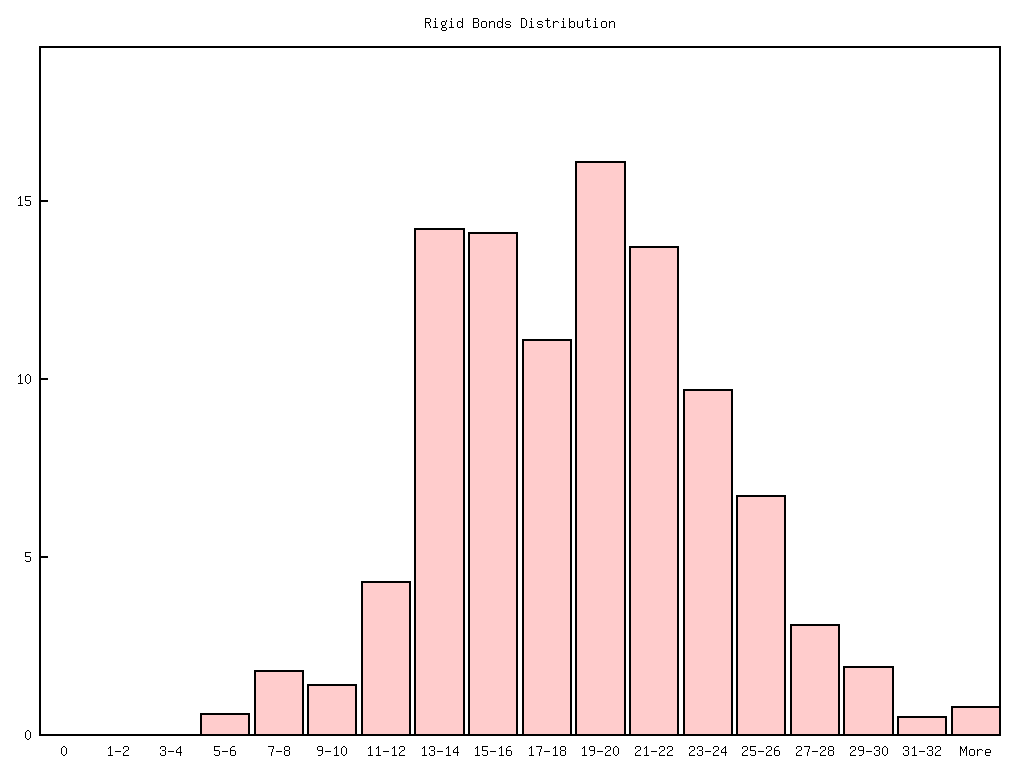

Supplement: Additional file 1 — This file contains the FAF-Drugs2 package and user manual. [file 1471-2105-9-396-S1.gz › FAFDrugs2/example/FAFDrugs2_OUTPUT_Jul_16_2008_17h57m34/RigidBondsDistribution.png]

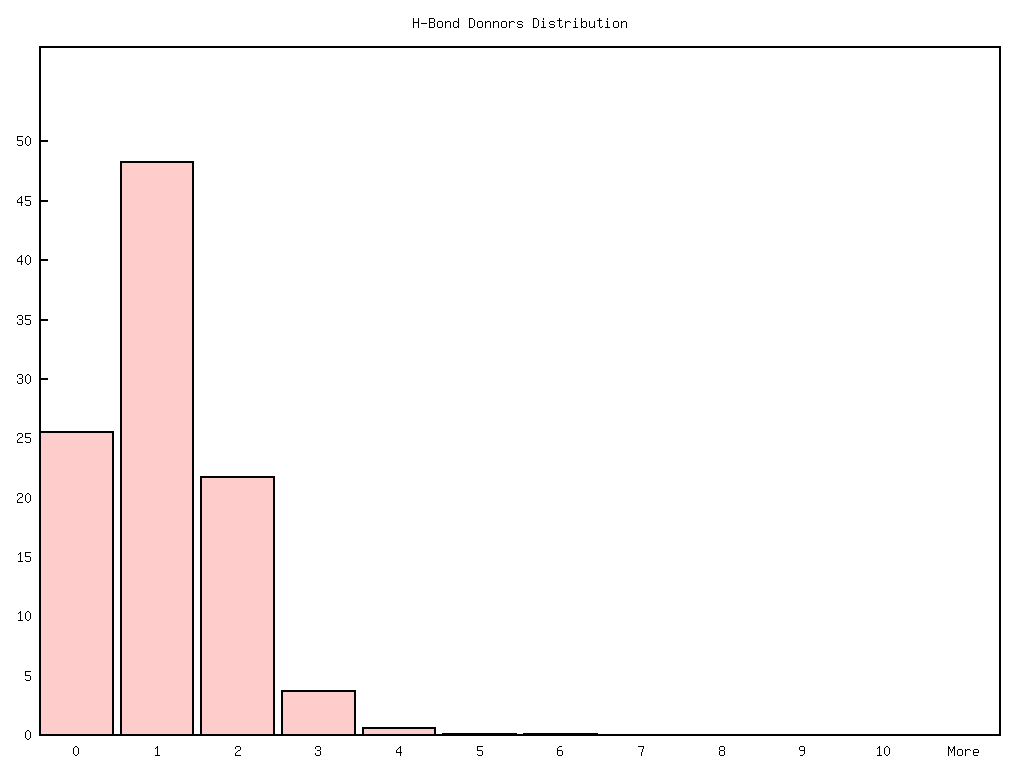

Supplement: Additional file 1 — This file contains the FAF-Drugs2 package and user manual. [file 1471-2105-9-396-S1.gz › FAFDrugs2/example/FAFDrugs2_OUTPUT_Jul_16_2008_17h57m34/HBondDonnorsDistribution.png]

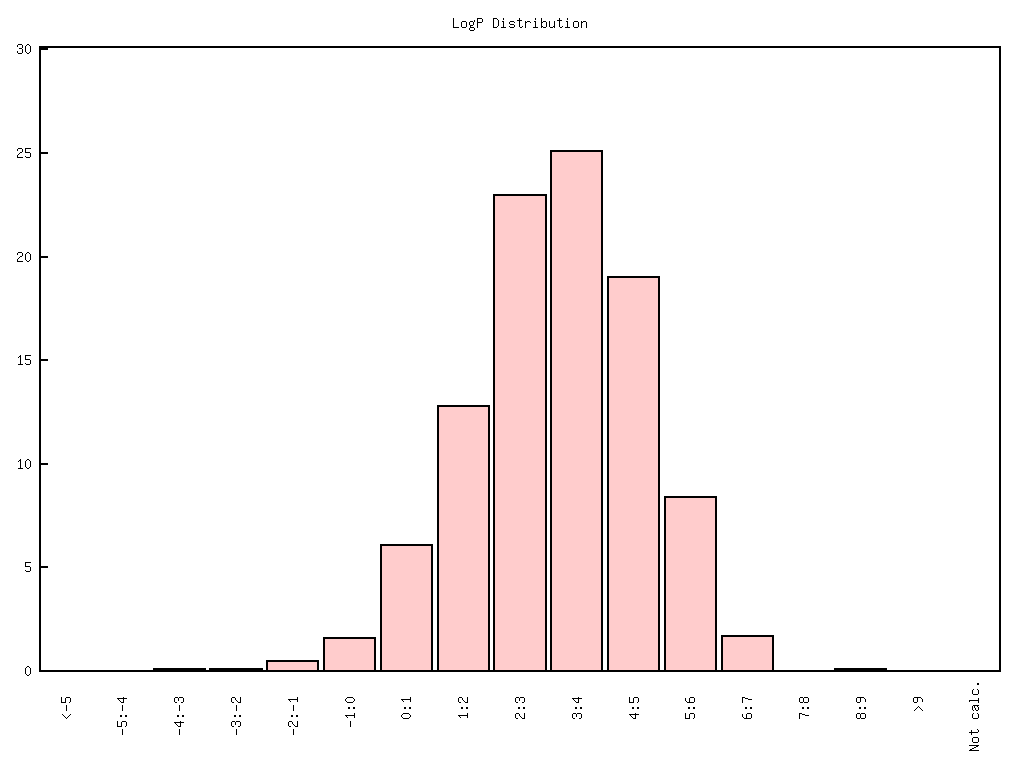

Supplement: Additional file 1 — This file contains the FAF-Drugs2 package and user manual. [file 1471-2105-9-396-S1.gz › FAFDrugs2/example/FAFDrugs2_OUTPUT_Jul_16_2008_17h57m34/LogPDistribution.png]

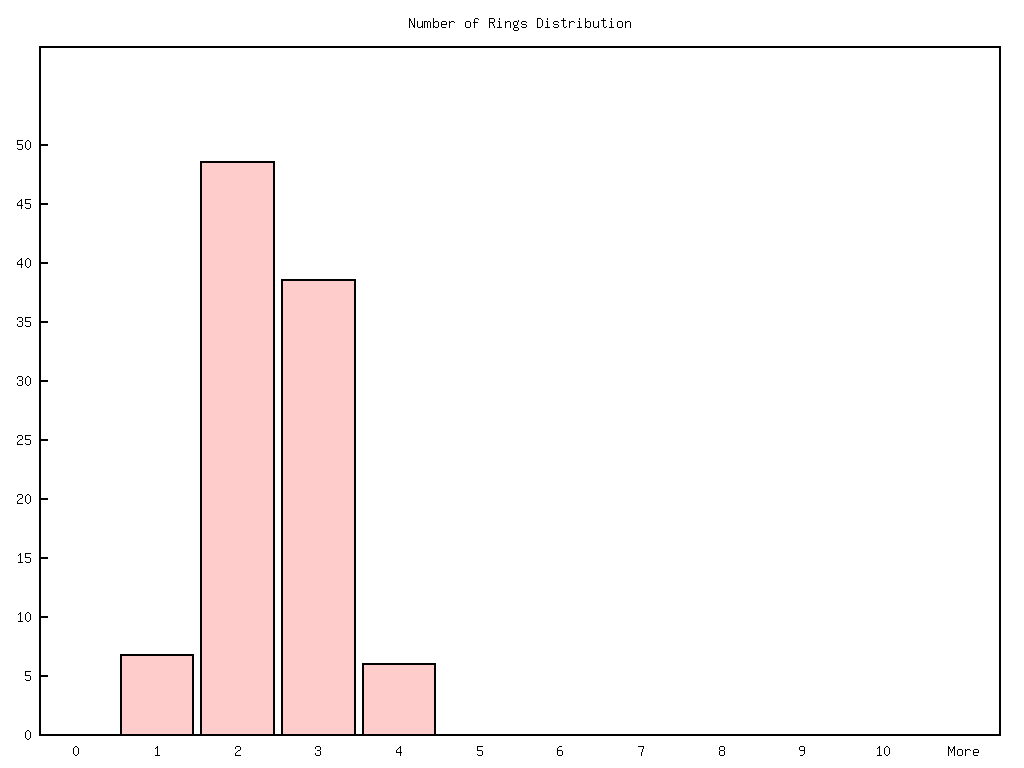

Supplement: Additional file 1 — This file contains the FAF-Drugs2 package and user manual. [file 1471-2105-9-396-S1.gz › FAFDrugs2/example/FAFDrugs2_OUTPUT_Jul_16_2008_17h57m34/RingsDistribution.png]

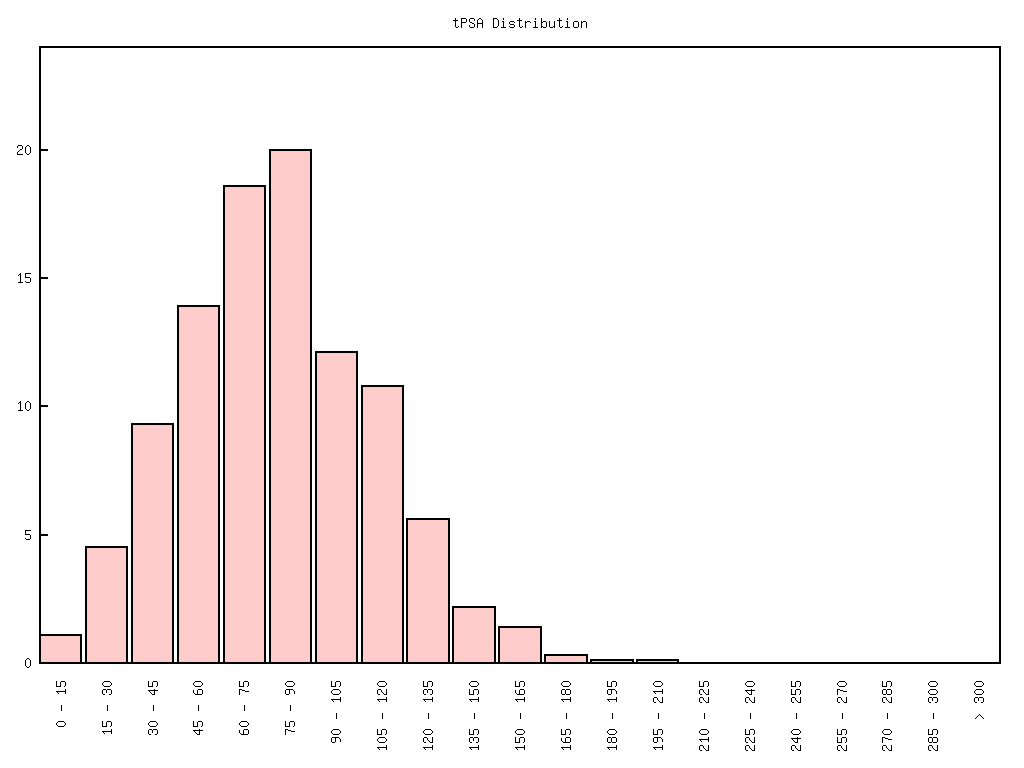

Supplement: Additional file 1 — This file contains the FAF-Drugs2 package and user manual. [file 1471-2105-9-396-S1.gz › FAFDrugs2/example/FAFDrugs2_OUTPUT_Jul_16_2008_17h57m34/tPSADistribution.png]

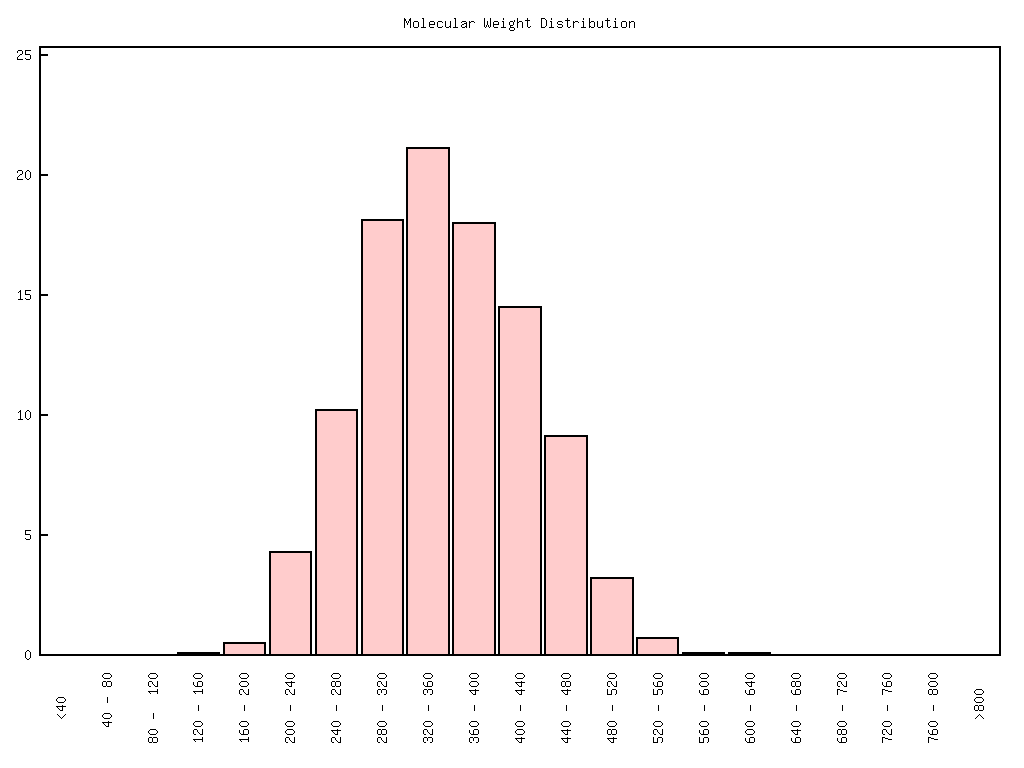

Supplement: Additional file 1 — This file contains the FAF-Drugs2 package and user manual. [file 1471-2105-9-396-S1.gz › FAFDrugs2/example/FAFDrugs2_OUTPUT_Jul_16_2008_17h57m34/MolecularWeightDistribution.png]

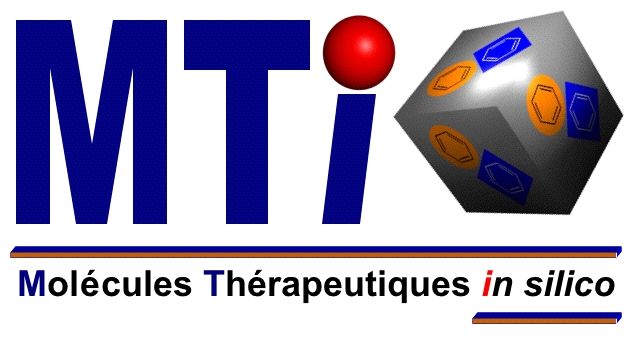

Supplement: Additional file 1 — This file contains the FAF-Drugs2 package and user manual. [file 1471-2105-9-396-S1.gz › FAFDrugs2/docs/UserGuide_FAF-Drugs2_fichiers/image001.jpg]

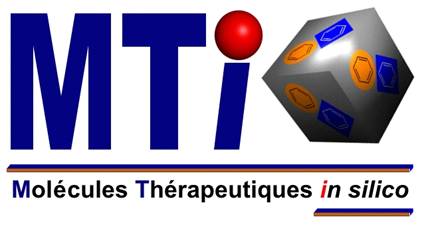

Supplement: Additional file 1 — This file contains the FAF-Drugs2 package and user manual. [file 1471-2105-9-396-S1.gz › FAFDrugs2/docs/UserGuide_FAF-Drugs2_fichiers/image002.jpg]
