# Supplementary material for: FAF-Drugs2: Free ADME/tox filtering tool to assist drug discovery and chemical biology projects
Source: BMC Bioinformatics. 2008 Sep 24;9:396. doi: 10.1186/1471-2105-9-396 (PMC2561050; doi:10.1186/1471-2105-9-396)
Supplement: Additional file 1 — This file contains the FAF-Drugs2 package and user manual. [file 1471-2105-9-396-S1.gz › FAFDrugs2/extras/openbabel-2.1.1.tar.gz!/openbabel-2.1.1/doc/babel.html]

babel

Table of Contents

## Name

**babel** -- a converter for chemistry and molecular modeling data files

## **Synopsis**

**babel** [**-H** *help-options*]  
**babel** [*OPTIONS*] [**-i** *input-type*] *infile* [**-o** *output-type*] *outfile*

## **Description**

Babel is a cross-platform program designed to interconvert between many
file formats used in molecular modeling and computational chemistry and
related areas.

Open Babel is also a complete programmers toolkit for developing chemistry
software. For more information, se the Open Babel web pages
<**http://openbabel.sourceforge.net/**>.

## **Options**

If only input and ouput files are given, Open Babel will guess the file
type from the filename extension.

**-a** *options*
:   Format-specific input options. See **-H** *format-ID* for options allowed
    by a particular format

**--addtotitle**
:   Append text to the current molecule title

**--addformula**
:   Append the molecular formula after the current molecule title

**-b**
:   Convert dative bonds: e.g., [N+]([O-])=O to N(=O)=O

**-c**
:   Center atomic coordinates at (0,0,0)

**-C**
:   Combine molecules in first file with others having the same name

**-e**
:   Continue after errors

**-d**
:   Delete Hydrogens

**---errorlevel** *2*
:   Filter the level of errors and warnings displayed:
    1 = critical errors only
    2 = include warnings too (default)
    3 = include informational messages too
    4 = include “audit log” messages of changes to data
    5 = include debugging messages too

**-f** *#*
:   For multiple entry input, start import with molecule # as the first
    entry

**-F**
:   Output the available fingerprint types

**-h**
:   Add hydrogens

**-H**
:   Output usage information

**-H** *format-ID*
:   Output formatting information and options for format specified

**-Hall**
:   Output formatting information and options for all formats

**-i<format-ID>**
:   Specifies input format, see below for the available formats

**-j**


**--join**
:   Join all input molecules into a single output molecule entry

**-k**
:   Translate computational chemistry modeling keywords (e.g., GAMESS
    and Gaussian)

**-m**
:   Produce multiple output files, to allow:

**-**
:   Splitting one input file - put each molecule into consecutively
    numbered output files

**-**
:   Batch conversion - convert each of multiple input files
    into a specified output format

**-l** *#*
:   For multiple entry input, stop import with molecule # as the last
    entry

**-o** *format-ID*
:   Specifies output format, see below for the available formats

**-p**
:   Add Hydrogens appropriate for pH (use transforms in phmodel.txt)

**--property**
:   Add or replace a property (e.g., in an MDL SD file)

**-s** *SMARTS*
:   Convert only molecules matching the SMARTS pattern specified

**--separate**
:   Separate disconnected fragments into individual molecular records

**-t**
:   All input files describe a single molecule

**--title** *title*
:   Add or replace molecular title

**-x** *options*
:   Format-specific output options. See **-H** *format-ID* for options
    allowed by a particular format

**-v** *SMARTS*
:   Convert only molecules **NOT** matching SMARTS pattern specified

**-V**
:   Output version number and exit

**-z**
:   Compress the output with gzip

## **File** **Formats**

The following formats are currently supported by Open Babel:

- acr -- Carine ASCI Crystal
- alc -- Alchemy format
- arc -- Accelrys/MSI Biosym/Insight II CAR format [Read-only]
- bgf -- MSI BGF format
- box -- Dock 3.5 Box format
- bs -- Ball and Stick format
- c3d1 -- Chem3D Cartesian 1 format
- c3d2 -- Chem3D Cartesian 2 format
- caccrt -- Cacao Cartesian format
- cache -- CAChe MolStruct format [Write-only]
- cacint -- Cacao Internal format [Write-only]
- can -- Canonical SMILES format
- car -- Accelrys/MSI Biosym/Insight II CAR format [Read-only]
- ccc -- CCC format [Read-only]
- cdx -- ChemDraw binary format [Read-only]
- cdxml -- ChemDraw CDXML format
- cht -- Chemtool format [Write-only]
- cif -- Crystallographic Information File
- cml -- Chemical Markup Language
- cmlr -- CML Reaction format
- com -- Gaussian 98/03 Cartesian Input [Write-only]
- copy -- Copies raw text [Write-only]
- crk2d -- Chemical Resource Kit 2D diagram format
- crk3d -- Chemical Resource Kit 3D format
- csr -- Accelrys/MSI Quanta CSR format [Write-only]
- cssr -- CSD CSSR format [Write-only]
- ct -- ChemDraw Connection Table format
- dmol -- DMol3 coordinates format
- ent -- Protein Data Bank format
- fa -- FASTA format [Write-only]
- fasta -- FASTA format [Write-only]
- fch -- Gaussian formatted checkpoint file format [Read-only]
- fchk -- Gaussian formatted checkpoint file format [Read-only]
- fck -- Gaussian formatted checkpoint file format [Read-only]
- feat -- Feature format
- fh -- Fenske-Hall Z-Matrix format [Write-only]
- fix -- SMILES FIX format [Write-only]
- fpt -- Fingerprint format [Write-only]
- fract -- Free Form Fractional format
- fs -- Open Babel FastSearching database
- fsa -- FASTA format [Write-only]
- g03 -- Gaussian 98/03 Output [Read-only]
- g98 -- Gaussian 98/03 Output [Read-only]
- gam -- GAMESS Output [Read-only]
- gamin -- GAMESS Input [Write-only]
- gamout -- GAMESS Output [Read-only]
- gau -- Gaussian 98/03 Cartesian Input [Write-only]
- gjc -- Gaussian 98/03 Cartesian Input [Write-only]
- gjf -- Gaussian 98/03 Cartesian Input [Write-only]
- gpr -- Ghemical format
- gr96 -- GROMOS96 format [Write-only]
- hin -- HyperChem HIN format
- inchi -- IUPAC InChI [Write-only]
- inp -- GAMESS Input [Write-only]
- ins -- ShelX format [Read-only]
- jin -- Jaguar input format [Write-only]
- jout -- Jaguar output format [Read-only]
- mdl -- MDL MOL format
- mmd -- MacroModel format
- mmod -- MacroModel format
- mol -- MDL MOL format
- mol2 -- Sybyl Mol2 format
- molreport -- Open Babel molecule report [Write-only]
- moo -- MOPAC Output format [Read-only]
- mop -- MOPAC Cartesian format
- mopcrt -- MOPAC Cartesian format
- mopin -- MOPAC Internal
- mopout -- MOPAC Output format [Read-only]
- mpc -- MOPAC Cartesian format
- mpd -- Sybyl descriptor format [Write-only]
- mpqc -- MPQC output format [Read-only]
- mpqcin -- MPQC simplified input format [Write-only]
- nw -- NWChem input format [Write-only]
- nwo -- NWChem output format [Read-only]
- pc -- PubChem format [Read-only]
- pcm -- PCModel format
- pdb -- Protein Data Bank format
- pov -- POV-Ray input format [Write-only]
- pqs -- Parallel Quantum Solutions format
- prep -- Amber Prep format [Read-only]
- qcin -- Q-Chem input format [Write-only]
- qcout -- Q-Chem output format [Read-only]
- report -- Open Babel report format [Write-only]
- res -- ShelX format [Read-only]
- rxn -- MDL RXN format
- sd -- MDL MOL format
- sdf -- MDL MOL format
- smi -- SMILES format
- sy2 -- Sybyl Mol2 format
- tdd -- Thermo format
- test -- Test format [Write-only]
- therm -- Thermo format
- tmol -- TurboMole Coordinate format
- txyz -- Tinker MM2 format [Write-only]
- unixyz -- UniChem XYZ format
- vmol -- ViewMol format
- xed -- XED format [Write-only]
- xml -- General XML format [Read-only]
- xyz -- XYZ cartesian coordinates format
- yob -- YASARA.org YOB format
- zin -- ZINDO input format [Write-only]

## **Format** **Options**

Individual file formats may have additional formatting options.

Input format options are preceded by ‘a’, e.g. `-as`

Output format options are preceded by ‘x’, e.g. `-xn`

For further specific information and options, use `-H<format-type>`
e.g., `-Hcml`

## **Examples**

Standard conversion:  

```
babel -ixyz ethanol.xyz -opdb ethanol.pdb
```

Conversion from a SMI file in STDIN to a Mol2 file written to STDOUT:  

```
babel -ismi -omol2
```

Split a multi-molecule file into new1.smi, new2.smi, etc.:  

```
babel infile.mol new.smi -m
```

## **See** **Also**

obenergy(1)
, obfit(1)
, obgrep(1)
, obminimize(1)
, obprop(1)
, obrotate(1)
, obrotamer(1)
.

The web pages for Open Babel can be found at:
<**http://openbabel.sourceforge.net/**>

More details on the formats supported by Open Babel can be found at:
<**http://openbabel.sourceforge.net/wiki/Formats**>

## **Authors**

A cast of many, including the currrent maintainers Geoff Hutchison, Chris
Morley, Michael Banck, and innumerable others who have contributed fixes
and additions. For more contributors to Open Babel, see <**http://openbabel.sourceforge.net/wiki/THANKS**>

## **Copyright**

Copyright (C) 1998-2001 by OpenEye Scientific Software, Inc.  
Some portions Copyright (C) 2001-2007 by Geoffrey R. Hutchison

This program is free software; you can redistribute it and/or modify it
under the terms of the GNU General Public License as published by the
Free Software Foundation version 2 of the License.

This program is distributed in the hope that it will be useful, but
WITHOUT ANY WARRANTY; without even the implied warranty of MERCHANTABILITY
or FITNESS FOR A PARTICULAR PURPOSE. See the GNU General Public
License for more details.

---

**Table of Contents**

- Name
- Synopsis
- Description
- Options
- File Formats
- Format Options
- Examples
- See Also
- Authors
- Copyright
