# Supplementary material for: FAF-Drugs2: Free ADME/tox filtering tool to assist drug discovery and chemical biology projects
Source: BMC Bioinformatics. 2008 Sep 24;9:396. doi: 10.1186/1471-2105-9-396 (PMC2561050; doi:10.1186/1471-2105-9-396)
Supplement: Additional file 1 — This file contains the FAF-Drugs2 package and user manual. [file 1471-2105-9-396-S1.gz › FAFDrugs2/extras/openbabel-2.1.1.tar.gz!/openbabel-2.1.1/doc/obfit.html]

obfit

Table of Contents

## Name

**obfit** -- superimpose two molecules based on a pattern

## **Synopsis**

**obfit** *SMARTS-pattern* *fixed-file* *outfile*

## **Description**

Superimpose two molecules using a quaternion fit. The atoms used to fit
the two molecules are defined by the SMARTS pattern given by the user. It
is useful to align congeneric series of molecules on a common structural
scaffold for 3D-QSAR studies. It can also be useful for displaying the
results of conformational generation.

Any molecules matching the supplied SMARTS pattern will be rotated and
translated to provide the smallest possible RMSD between the matching
regions. If a molecule does not match the SMARTS pattern, it will be output
with no transformation.

## **Examples**

Align all the molecules in ‘testmv.sdf’ on a single molecule of
‘testref.sdf’ by superimposing them on its N-methylpiperidyl portion (and outputting a new SD file to the standard output):

```
obfit '[nh]1c2c(=O)n(C)c(=O)n(C)c2cc1' testref.sdf testmv.sdf
```

## **See** **Also**

babel(1)
, obchiral(1)
, obfit(1)
, obgrep(1)
, obrotate(1)
.

The web pages for Open Babel can be found at: <**http://openbabel.sourceforge.net/**>

A guide for constructing SMARTS patterns can be found at:
<**http://www.daylight.com/dayhtml/doc/theory/theory.smarts.html**>

## **Authors**

The obfit program was contributed by Fabien Fontaine

Open Babel is developed by a cast of many, including currrent maintainers
Geoff Hutchison, Chris Morley, Michael Banck, and innumerable others who
have contributed fixes and additions. For more contributors to Open
Babel, see <**http://openbabel.sourceforge.net/wiki/THANKS**>

## **Copyright**

Copyright (C) 1998-2001 by OpenEye Scientific Software, Inc.  
Some portions Copyright (C) 2001-2007 by Geoffrey R. Hutchison and other
contributors.

This program is free software; you can redistribute it and/or modify it
under the terms of the GNU General Public License as published by the
Free Software Foundation version 2 of the License.

This program is distributed in the hope that it will be useful, but
WITHOUT ANY WARRANTY; without even the implied warranty of MERCHANTABILITY
or FITNESS FOR A PARTICULAR PURPOSE. See the GNU General Public
License for more details.

---

**Table of Contents**

- Name
- Synopsis
- Description
- Examples
- See Also
- Authors
- Copyright
