# Supplementary material for: FAF-Drugs2: Free ADME/tox filtering tool to assist drug discovery and chemical biology projects
Source: BMC Bioinformatics. 2008 Sep 24;9:396. doi: 10.1186/1471-2105-9-396 (PMC2561050; doi:10.1186/1471-2105-9-396)
Supplement: Additional file 1 — This file contains the FAF-Drugs2 package and user manual. [file 1471-2105-9-396-S1.gz › FAFDrugs2/extras/openbabel-2.1.1.tar.gz!/openbabel-2.1.1/doc/obgrep.html]

obgrep

Table of Contents

## Name

**obgrep** -- an advanced molecular search program using SMARTS

## **Synopsis**

**obgrep** [*OPTIONS*] *‘SMARTS-pattern’* *filename*

## **Description**

The obgrep tool can be used to search for molecules inside multi-molecule
database files (e.g., SMILES, SDF, etc.) or across multiple files.

## **Options**

If only a filename is given, obgrep will attempt to guess the file type
from the filename extension.

**-c**
:   Print the number of matches

**-f**
:   Full match, print matching-molecules only when the number of heavy
    atoms is also equal to the number of atoms in the SMARTS pattern

**-i** *format*
:   Specifies input and output format, see babel(1)
    for available formats

**-n**
:   Only print the name of the molecules

**-t** *#*
:   Print a molecule only if the pattern occurs # times inside the molecule

**-v**
:   Invert the matching, print non-matching molecules

## **Examples**

Note that in all examples, the SMARTS pattern is enclosed in single
quotes '...' to ensure it is not changed by the shell.

Print all the molecules with a methylamine group:  

```
obgrep 'CN' database.smi
```

Print all the molecules without a methylamine group:  

```
obgrep -v 'CN' database.smi
```

Print the number of molecules without a methylamine group:  

```
obgrep -v -c 'CN' database.smi
```

Print methylamine (if it exists in the file):  

```
obgrep -f 'CN' database.smi
```

Print methylamine and/or methanol (if they exist):  

```
obgrep -f 'C[N,O]' database.smi
```

Print all molecules with aromatic carbon in all SMILES files in the
directory (i.e., \*.smi):  

```
obgrep 'c' *.smi
```

## **See** **Also**

babel(1)
, obfit(1)
, obrotate(1)
.

The web pages for Open Babel can be found at: <**http://openbabel.sourceforge.net/**>

A guide for constructing SMARTS patterns can be found at:
<**http://www.daylight.com/dayhtml/doc/theory/theory.smarts.html**>

## **Authors**

The obgrep program was contributed by Fabien Fontaine

Open Babel is developed by a cast of many, including currrent maintainers
Geoff Hutchison, Chris Morley, Michael Banck, and innumerable others who
have contributed fixes and additions. For more contributors to Open
Babel, see <**http://openbabel.sourceforge.net/wiki/THANKS**>

## **Copyright**

Copyright (C) 1998-2001 by OpenEye Scientific Software, Inc.  
Some portions Copyright (C) 2001-2007 by Geoffrey R. Hutchison and other
contributors.

This program is free software; you can redistribute it and/or modify it
under the terms of the GNU General Public License as published by the
Free Software Foundation version 2 of the License.

This program is distributed in the hope that it will be useful, but
WITHOUT ANY WARRANTY; without even the implied warranty of MERCHANTABILITY
or FITNESS FOR A PARTICULAR PURPOSE. See the GNU General Public
License for more details.

---

**Table of Contents**

- Name
- Synopsis
- Description
- Options
- Examples
- See Also
- Authors
- Copyright
