# Supplementary material for: FAF-Drugs2: Free ADME/tox filtering tool to assist drug discovery and chemical biology projects
Source: BMC Bioinformatics. 2008 Sep 24;9:396. doi: 10.1186/1471-2105-9-396 (PMC2561050; doi:10.1186/1471-2105-9-396)
Supplement: Additional file 1 — This file contains the FAF-Drugs2 package and user manual. [file 1471-2105-9-396-S1.gz › FAFDrugs2/extras/openbabel-2.1.1.tar.gz!/openbabel-2.1.1/doc/obprop.html]

obprop

Table of Contents

## Name

**obprop** -- print standard molecular properties

## **Synopsis**

**obprop** *filename*

## **Description**

The obprop program is a tool to print a set of standard molecular properties
for all molecules in a file. It also serves as example code for
using the Open Babel library (libopenbabel).

Output format includes:  
name [*Name*]  
formula [*Formula*]  
mol\_weight [*Molecular* *Weight*]  
exact\_mass [*Isotopic* *Mass*]  
canonical\_SMILES [*String*]  
num\_atoms [*Number*]  
num\_bonds [*Number*]  
num\_residues [*Number*]  
sequence [*Residue* *Sequence*]  
num\_rings [*Number* *of* *Rings* *(by* *SSSR)*]  
logP [*Number* *(octanol-water* *partition)*]  
PSA [*Number* *(topological* *polar* *surface* *area)*]
MR [*Number* *(molar* *refractivity)*]  
$$$

The “$$$” is the separator between multiple molecular entries in a file

## **Examples**

```
obprop pyridines.sdf
```

## **See** **Also**

babel(1)
, obchiral(1)
, obfit(1)
, obgrep(1)
, obrotate(1)
.

The web pages for Open Babel can be found at: <**http://openbabel.sourceforge.net/**>

## **Authors**

The obprop program was contributed by Fabien Fontaine

Open Babel is developed by a cast of many, including currrent maintainers
Geoff Hutchison, Chris Morley, Michael Banck, and innumerable others who
have contributed fixes and additions. For more contributors to Open
Babel, see <**http://openbabel.sourceforge.net/wiki/THANKS**>

## **Copyright**

Copyright (C) 2003 by Fabien Fontaine  
Some portions Copyright (C) 2004-2007 by Geoffrey R. Hutchison and other
contributors.

This program is free software; you can redistribute it and/or modify it
under the terms of the GNU General Public License as published by the
Free Software Foundation version 2 of the License.

This program is distributed in the hope that it will be useful, but
WITHOUT ANY WARRANTY; without even the implied warranty of MERCHANTABILITY
or FITNESS FOR A PARTICULAR PURPOSE. See the GNU General Public
License for more details.

---

**Table of Contents**

- Name
- Synopsis
- Description
- Examples
- See Also
- Authors
- Copyright
