# Supplementary material for: FAF-Drugs2: Free ADME/tox filtering tool to assist drug discovery and chemical biology projects
Source: BMC Bioinformatics. 2008 Sep 24;9:396. doi: 10.1186/1471-2105-9-396 (PMC2561050; doi:10.1186/1471-2105-9-396)
Supplement: Additional file 1 — This file contains the FAF-Drugs2 package and user manual. [file 1471-2105-9-396-S1.gz › FAFDrugs2/extras/openbabel-2.1.1.tar.gz!/openbabel-2.1.1/doc/obrotate.html]

obrotate

Table of Contents

## Name

**obrotate** -- batch-rotate dihedral angles matching SMARTS patterns

## **Synopsis**

**obrotate** *‘SMARTS-pattern’* *filename* *atom1* *atom2* *atom3* *atom4* *angle*

## **Description**

The obrotate program rotates the torsional (dihedral) angle of a specified
bond in molecules to that defined by the user. In other words, it
does the same as a user setting an angle in a molecular modelling package,
but much faster and in batch mode (i.e. across multiple molecules in
a file).

The four atom IDs required are indexes into the SMARTS pattern, which
starts at atom 0 (zero). The angle supplied is in degrees. The two atoms
used to set the dihedral angle <atom1> and <atom4> do not need to be connected
to the atoms of the bond <atom2> and <atom3> in any way.

The order of the atoms matters -- the portion of the molecule attached to
<atom1> and <atom2> remain fixed, but the portion bonded to <atom3> and &
<atom4> moves.

## **Examples**

Let’s say that you want to define the conformation of a large number of
molecules with a pyridyl scaffold and substituted with an aliphatic chain
at the 3-position, for example for docking or 3D-QSAR purposes.

To set the value of the first dihedral angle to 90 degrees:  

```
obrotate ‘c1ccncc1CCC’ pyridines.sdf 5 6 7 8 90
```

Here 6 and 7 define the bond to rotate in the SMARTS patter, i.e., c1-C
and atoms 5 and 8 define the particular dihedral angle to rotate.

Since the atoms to define the dihedral do not need to be directly connected,
the nitrogen in the pyridine can be used:  

```
obrotate ‘c1ccncc1CCC’ pyridines.sdf 4 6 7 8 90
```

Keep the pyridyl ring fixed and moves the aliphatic chain:  

```
obrotate ‘c1ccncc1CCC’ pyridines.sdf 5 6 7 8 90
```

Keep the aliphatic chain fixed and move the pyridyl ring:  

```
obrotate ‘c1ccncc1CCC’ pyridines.sdf 8 7 6 5 90
```

## **See** **Also**

babel(1)
, obchiral(1)
, obfit(1)
, obgrep(1)
, obprop(1)
, obrotate(1)
.

The web pages for Open Babel can be found at: <**http://openbabel.sourceforge.net/**>

A guide for constructing SMARTS patterns can be found at:
<**http://www.daylight.com/dayhtml/doc/theory/theory.smarts.html**>

## **Authors**

The obgrep program was contributed by Fabien Fontaine

Open Babel is developed by a cast of many, including currrent maintainers
Geoff Hutchison, Chris Morley, Michael Banck, and innumerable others who
have contributed fixes and additions. For more contributors to Open
Babel, see <**http://openbabel.sourceforge.net/wiki/THANKS**>

## **Copyright**

Copyright (C) 1998-2001 by OpenEye Scientific Software, Inc.  
Some portions Copyright (C) 2001-2007 by Geoffrey R. Hutchison and other
contributors.

This program is free software; you can redistribute it and/or modify it
under the terms of the GNU General Public License as published by the
Free Software Foundation version 2 of the License.

This program is distributed in the hope that it will be useful, but
WITHOUT ANY WARRANTY; without even the implied warranty of MERCHANTABILITY
or FITNESS FOR A PARTICULAR PURPOSE. See the GNU General Public
License for more details.

---

**Table of Contents**

- Name
- Synopsis
- Description
- Examples
- See Also
- Authors
- Copyright
