# Supplementary material for: FAF-Drugs2: Free ADME/tox filtering tool to assist drug discovery and chemical biology projects
Source: BMC Bioinformatics. 2008 Sep 24;9:396. doi: 10.1186/1471-2105-9-396 (PMC2561050; doi:10.1186/1471-2105-9-396)
Supplement: Additional file 1 — This file contains the FAF-Drugs2 package and user manual. [file 1471-2105-9-396-S1.gz › FAFDrugs2/extras/openbabel-2.1.1.tar.gz!/openbabel-2.1.1/doc/OBTwoMigration.html]

Open Babel: Migration from 1.x to 2.0


# Migration from Open Babel 1.x

## (a.k.a. What's New for Developers in Open Babel 2.0)

$Date: 2005-11-17 19:53:15 -0500 (Thu, 17 Nov 2005) $

## General:

Many things have changed internally in the new Open Babel 2.0
release, including a variety of new classes, methods, and
algorithms. For a full list of what's new, see the Release Notes and Change Log.

This document aims to outline major changes in the Open Babel
library which differ from previous versions. In general, major version numbers of Open Babel indicate major changes to the library which will break backwards-compatibility in at least some areas. For example 1.x to 2.x represents changes that are backwards-incompatible as well as several new interfaces. Changes between 2.0 and 2.1 will **add** but **not break** existing code

There are four main areas of updates:

- OBFileFormat vs. OBConversion
- Iterator Classes
- Error Handling
- Generic Data

## OBFileFormat vs. OBConversion

By far the largest change between Open Babel 1.x and 2.0 is the
introduction of the OBConversion and OBFormat classes to handle
reading and writing chemical data, replacing the OBFileFormat
class. There are several main advantages to these new classes.

- Dynamic loading and unloading of file formats.

- This means that to write a new format, only one file (with the
  format code) is needed. No other code changes are required.
- In Open Babel 1.x, in addition to the file format code, 2 code
  files, 2 header files, and extable.txt needed to be changed.

- Better support for formats which handle multiple molecules "records" in one file (e.g., CML, MDL Molfile, SMILES, etc.).
- Batch conversion, splitting, and joining multiple molecule files.
- Support for handling reaction data (e.g., MDL Rxn, CMLReact files) and other types of chemical data beyond simple molecular files.

### Code Changes

Example code for accessing OBConversion and OBFormat to translate files is included in all of the command-line programs `babel`, `obgrep`, `obfit`, etc. For example:

```
      // old code
      ifstream ifs(filename);
 
      io_type inFileType = extab.FilenameToType(FileIn);

      if (extab.IsReadable(inFileType) && extab.IsWritable(SMI)
      {
          OBMol mol(inFileType, SMI);
          stringstream outstream;
          fileFormat.ReadMolecule(ifs, mol, filename);
          fileFormat.WriteMolecule(outstream, mol);
          ...
      }
```

now becomes

```
        // new code
        ifstream ifs(filename);
        
        OBConversion conv;
        // Try 
        OBFormat* inFormat = conv.FormatFromExt(filename);
        OBFormat* outFormat = conv.GetFormat("SMI");
        istream* pIn = &ifs 
        stringstream newstream;
        if(inFormat && outFormat)
        {
                conv.SetInAndOutFormats(inFormat,outFormat);
                conv.Convert(pIn,&newstream);   // note works on more than just OBMol objects! (reactions...)
                ...
        }
```

Here's another example of setting up the OBConversion framework:

```
        OBConversion conv(&cin,&cout);
        if(conv.SetInAndOutFormats("SMI","MOL"))
        {       
                OBMol mol;
                if(conv.Read(&mol))
                ...manipulate molecule 
                
                conv->Write(&mol);
        }
```

Some small changes are needed to the file format translator code modules themselves. In general, these are easy to see from the current code files, e.g., `src/formats/xyzformat.cpp`. If your format writes molecular records (as opposed to reactions), you will likely be able to derive from the OBMoleculeFormat class.

For more information, see the OBConversion API documentation.

Top

## Iterator Classes

To facilitate iteration through all atoms, bonds, residues, etc, without resorting to index access (which may change in the future) or the various OBMol::BeginAtom() and OBAtom::NextAtom() methods which may only be safely used by one method at once (e.g., if your code is multithreaded, or a method above your code or underneath your code uses these iterators, errors will occur).

Therefore, it is **highly recommended** to use the new STL-style iterator classes introduced into Open Babel 2.0.

### Code Changes

No old code **needs** to be updated to work with Open Babel 2.0. However, the old iterator methods are deprecated and will disappear in some future release. The new methods are easier to use and less error-prone, so it is highly recommended to convert. For example:

```
   // old code
   #include "mol.h"

   OBAtom *atom;
   OBAtom *nbr;
   vector::iterator i;

   for (nbr = atom->BeginNbrAtom(i);nbr;nbr = atom->NextNbrAtom(i))
...
```

becomes

```
   // new code
   #include "obiter.h"
   #include "mol.h"

   OBAtom *atom;
   FOR_NBORS_OF_ATOM(nbr, atom)
    {
         ...
```

For more information, see the documentation for the OBMol class.

Top

## Error Handling

In order to allow users and developers to easily redirect, filter, and log errors, debugging messages, and internal "audit log" information when molecules are altered, the new OBMessageHandler class has been added.

### Code Changes

Rather than using `std::cerr` or `std::cout` or the old **ThrowError()** method, you should use the global obErrorLog object.

```
   ThrowError("Requested Atom Out of Range");
 ...
 
   std::cerr << " Could not parse line in type translation table types.txt -- incorect number of columns";
   std::cerr << " found " << vc.size() << " expected " << _ncols << "." << std::endl;
```

becomes...

```
    // new code
   obErrorLog.ThrowError(__FUNCTION__, "Requested Atom Out of Range", obDebug);

...

     stringstream errorMsg;
     errorMsg << " Could not parse line in type translation table types.txt -- incorect number of columns";
     errorMsg << " found " << vc.size() << " expected " << _ncols << ".";
     obErrorLog.ThrowError(__FUNCTION__, errorMsg.str(), obInfo);
```

For more information, see the OBMessageHandler class.

Top

## Generic Data

The OBGenericData class has some small modifications, notably the expansion of hash-index access through the old obDataType class to OBGenericDataType, with named unsigned integers. In particular, this greatly facilitates the storage and manipulation of essentially an unlimited number of data types on a per-atom, per-bond, per-residue, or per-molecule basis.

### Code Changes

Very little needs to be done. If you have derived a subclass of OBGenericData, then you should also pick a new OBGenericDataType, using some of the undefined namespace. (We would prefer if you also let us know, so we can minimize conflicts or future compatibility problems.)

```
    if (mol.HasData(obUnitCell))
    {
        OBUnitCell *uc = (OBUnitCell*)mol.GetData(obUnitCell)
        ...
    }

    ...

    if (mol.HasData("Author"))
      ...
```

becomes...

```
    if (mol.HasData(OBGenericDataType::UnitCell))
    {
        OBUnitCell *uc = (OBUnitCell*)mol.GetData(OBGenericDataType::UnitCell)
        ...
    }

    // String access doesn't need to be changed at all
    if (mol.HasData("Author"))
      ...
```

Top
