# Supplementary material for: FAF-Drugs2: Free ADME/tox filtering tool to assist drug discovery and chemical biology projects
Source: BMC Bioinformatics. 2008 Sep 24;9:396. doi: 10.1186/1471-2105-9-396 (PMC2561050; doi:10.1186/1471-2105-9-396)
Supplement: Additional file 1 — This file contains the FAF-Drugs2 package and user manual. [file 1471-2105-9-396-S1.gz › FAFDrugs2/extras/openbabel-2.1.1.tar.gz!/openbabel-2.1.1/scripts/python/Examples.html]

Open Babel: Python Scripting


# Using the openbabel module with Python

The `openbabel` module is designed to allow Python scripts to use the C++ Open Babel library. The wrapper is generated using the SWIG package and provides access to almost all of the Open Babel interfaces via Python, including the base
classes OBMol, OBAtom, OBBond, and OBResidue, as well as the conversion framework OBConversion.

As such, essentially any call in the C++ API is available to Python scripts with very little difference in syntax. This guide is designed to give examples of common Python syntax for the `openbabel` module and pointers to the appropriate sections of the API documentation.

The example script below creates atoms and bonds one-by-one using the OBMol, OBAtom, and OBBond classes.

```
import openbabel

mol = openbabel.OBMol()
print 'Should print 0 (atoms)'
print mol.NumAtoms()

a = mol.NewAtom()
a.SetAtomicNum(6)   # carbon atom
a.SetVector(0.0, 1.0, 2.0) # coordinates

b = mol.NewAtom()
mol.AddBond(1, 2, 1)   # atoms indexed from 1
print 'Should print 2 (atoms)'
print mol.NumAtoms()
print 'Should print 1 (bond)'
print mol.NumBonds()

mol.Clear();
```

More commonly, Open Babel can be used to read in molecules using the OBConversion framework. The following script reads in molecular information (a SMI file) from a string, adds hydrogens, and writes out an MDL file as a string.

```
import openbabel

obConversion = openbabel.OBConversion()
obConversion.SetInAndOutFormats("smi", "mdl")

obConversion.ReadString(mol, "C1=CC=CS1")

print 'Should print 5 (atoms)'
print mol.NumAtoms()

mol.AddHydrogens()
print 'Should print 9 (atoms) after adding hydrogens'
print mol.NumAtoms()

outMDL = obConversion.WriteString(mol)
```

The following script writes out a file using a filename, rather than reading and writing to a Python string.

```
import openbabel

obConversion = openbabel.OBConversion()
obConversion.SetInAndOutFormats("pdb", "mol2")

obConversion.ReadFile(mol, "1ABC.pdb.gz")   # Open Babel will uncompress automatically

mol.AddHydrogens()

print mol.NumAtoms()
print mol.NumBonds()
print mol.NumResidues()

obConversion.WriteFile(mol, '1abc.mol2')
```

That's it! There's more information on particular calls in the library API. Feel free to address questions to the openbabel-scripting mailing list.
