# Supplementary material for: FAF-Drugs2: Free ADME/tox filtering tool to assist drug discovery and chemical biology projects
Source: BMC Bioinformatics. 2008 Sep 24;9:396. doi: 10.1186/1471-2105-9-396 (PMC2561050; doi:10.1186/1471-2105-9-396)
Supplement: Additional file 1 — This file contains the FAF-Drugs2 package and user manual. [file 1471-2105-9-396-S1.gz › FAFDrugs2/extras/openbabel-2.1.1.tar.gz!/openbabel-2.1.1/scripts/python/pybelapi.html]

Python: module pybel

|  |  |
| --- | --- |
| **pybel** | index /home/user/Tools/openbabel-2-1-xz/scripts/python/pybel.py |

**Modules**

|  |  |  |  |
| --- | --- | --- | --- |
| openbabel | os |  |  |

**Classes**

\_\_builtin\_\_.object
:   Atom

    Fingerprint

    Molecule

    MoleculeData

    Outputfile

    Smarts

|  |  |  |
| --- | --- | --- |
| class **Atom**(\_\_builtin\_\_.object) | | |
|  | Represent a Pybel atom.     Optional parameters:     OBAtom -- an Open Babel Atom (default is None)     index -- the index of the atom in the molecule (default is None)     An empty Atom is created if an Open Babel atom is not provided.     Attributes:     atomicmass, atomicnum, cidx, coords, coordidx, exactmass,     formalcharge, heavyvalence, heterovalence, hyb, idx,     implicitvalence, index, isotope, partialcharge, spin, type,     valence, vector.     (refer to the Open Babel library documentation for more info).     The original Open Babel atom can be accessed using the attribute:     OBAtom | |
|  |  | Methods defined here:  **\_\_getattr\_\_**(self, attr)  **\_\_init\_\_**(self, OBAtom=None, index=None)  **\_\_str\_\_**(self)  Create a string representation of the atom.     >>> a = Atom()  >>> print a  Atom: 0 (0.0, 0.0, 0.0)   ---  Data and other attributes defined here:  **\_\_dict\_\_** = <dictproxy object> dictionary for instance variables (if defined)  **\_\_weakref\_\_** = <attribute '\_\_weakref\_\_' of 'Atom' objects> list of weak references to the object (if defined) |

|  |  |  |
| --- | --- | --- |
| class **Fingerprint**(\_\_builtin\_\_.object) | | |
|  | A Molecular Fingerprint.     Required parameters:     obFingerprint -- a vector calculated by OBFingerprint.FindFingerprint()     Attributes:     fp -- the original obFingerprint     bits -- a list of bits set in the Fingerprint     Methods:     The "\|" operator can be used to calculate the Tanimoto coeff. For example,     given two Fingerprints 'a', and 'b', the Tanimoto coefficient is given by:        tanimoto = a \| b | |
|  |  | Methods defined here:  **\_\_getattr\_\_**(self, attr)  **\_\_init\_\_**(self, obFingerprint)  **\_\_or\_\_**(self, other)  **\_\_str\_\_**(self)   ---  Data and other attributes defined here:  **\_\_dict\_\_** = <dictproxy object> dictionary for instance variables (if defined)  **\_\_weakref\_\_** = <attribute '\_\_weakref\_\_' of 'Fingerprint' objects> list of weak references to the object (if defined) |

|  |  |  |
| --- | --- | --- |
| class **Molecule**(\_\_builtin\_\_.object) | | |
|  | Represent a Pybel molecule.     Optional parameters:     OBMol -- an Open Babel molecule (default is None)     An empty Molecule is created if an Open Babel molecule is not provided.     Attributes:     atoms, charge, data, dim, energy, exactmass, flags, formula,      mod, molwt, spin, sssr, title, unitcell.  (refer to the Open Babel library documentation for more info).     Methods:     write(), calcfp(), calcdesc()      The original Open Babel molecule can be accessed using the attribute:     OBMol | |
|  |  | Methods defined here:  **\_\_getattr\_\_**(self, attr)  Return the value of an attribute     Note: The values are calculated on-the-fly. You may want to store the value in  a variable if you repeatedly access the same attribute.  **\_\_init\_\_**(self, OBMol=None)  **\_\_iter\_\_**(self)  Iterate over the Atoms of the Molecule.     This allows constructions such as the following:     for atom in mymol:         print atom  **\_\_str\_\_**(self)  **calcdesc**(self, descnames=[])  Calculate descriptor values.     Optional parameter:     descnames -- a list of names of descriptors     If descnames is not specified, the full list of Open Babel  descriptors is calculated: LogP, PSA and MR.  **calcfp**(self, fptype='')  Calculate a molecular fingerprint.     Optional parameters:     fptype -- the name of the Open Babel fingerprint type.     If fptype is not specified, the default Open Babel fingerprint  type is used. See the Open Babel library documentation for more  details.  **write**(self, format='SMI', filename=None, overwrite=False)  Write the molecule to a file or return a string.     Optional parameters:     format -- default is "SMI"     filename -- default is None     overwite -- default is False     If a filename is specified, the result is written to a file.  Otherwise, a string is returned containing the result.  The overwrite flag is ignored if a filename is not specified.  It controls whether to overwrite an existing file.   ---  Data and other attributes defined here:  **\_\_dict\_\_** = <dictproxy object> dictionary for instance variables (if defined)  **\_\_weakref\_\_** = <attribute '\_\_weakref\_\_' of 'Molecule' objects> list of weak references to the object (if defined) |

|  |  |  |
| --- | --- | --- |
| class **MoleculeData**(\_\_builtin\_\_.object) | | |
|  | Store molecule data in a dictionary-type object     Required parameters:    obmol -- an Open Babel OBMol      Methods and accessor methods are like those of a dictionary except  that the data is retrieved on-the-fly from the underlying OBMol.     Example:  >>> mol = readfile("sdf", 'head.sdf').next()  >>> data = mol.data  >>> print data  {'Comment': 'CORINA 2.61 0041  25.10.2001', 'NSC': '1'}  >>> print len(data), data.keys(), data.has\_key("NSC")  2 ['Comment', 'NSC'] True  >>> print data['Comment']  CORINA 2.61 0041  25.10.2001  >>> data['Comment'] = 'This is a new comment'  >>> for k,v in data.iteritems():  ...    print k, "-->", v  Comment --> This is a new comment  NSC --> 1  >>> del data['NSC']  >>> print len(data), data.keys(), data.has\_key("NSC")  1 ['Comment'] False | |
|  |  | Methods defined here:  **\_\_contains\_\_**(self, key)  **\_\_delitem\_\_**(self, key)  **\_\_getitem\_\_**(self, key)  **\_\_init\_\_**(self, obmol)  **\_\_iter\_\_**(self)  **\_\_len\_\_**(self)  **\_\_repr\_\_**(self)  **\_\_setitem\_\_**(self, key, value)  **clear**(self)  **has\_key**(self, key)  **items**(self)  **iteritems**(self)  **keys**(self)  **update**(self, dictionary)  **values**(self)   ---  Data and other attributes defined here:  **\_\_dict\_\_** = <dictproxy object> dictionary for instance variables (if defined)  **\_\_weakref\_\_** = <attribute '\_\_weakref\_\_' of 'MoleculeData' objects> list of weak references to the object (if defined) |

|  |  |  |
| --- | --- | --- |
| class **Outputfile**(\_\_builtin\_\_.object) | | |
|  | Represent a file to which \*output\* is to be sent.     Although it's possible to write a single molecule to a file by  calling the write() method of a molecule, if multiple molecules  are to be written to the same file you should use the Outputfile  class.     Required parameters:     format     filename  Optional parameters:     overwrite (default is False) -- if the output file already exists,                                     should it be overwritten?  Methods:     write(molecule) | |
|  |  | Methods defined here:  **\_\_init\_\_**(self, format, filename, overwrite=False)  **close**(self)  Close the Outputfile to further writing.  **write**(self, molecule)  Write a molecule to the output file.     Required parameters:     molecule   ---  Data and other attributes defined here:  **\_\_dict\_\_** = <dictproxy object> dictionary for instance variables (if defined)  **\_\_weakref\_\_** = <attribute '\_\_weakref\_\_' of 'Outputfile' objects> list of weak references to the object (if defined) |

|  |  |  |
| --- | --- | --- |
| class **Smarts**(\_\_builtin\_\_.object) | | |
|  | A Smarts Pattern Matcher     Required parameters:     smartspattern     Methods:     findall()     Example:  >>> mol = readstring("smi","CCN(CC)CC") # triethylamine  >>> smarts = Smarts("[#6][#6]") # Matches an ethyl group  >>> print smarts.findall(mol)   [(1, 2), (4, 5), (6, 7)] | |
|  |  | Methods defined here:  **\_\_init\_\_**(self, smartspattern)  Initialise with a SMARTS pattern.  **findall**(self, molecule)  Find all matches of the SMARTS pattern to a particular molecule.     Required parameters:     molecule   ---  Data and other attributes defined here:  **\_\_dict\_\_** = <dictproxy object> dictionary for instance variables (if defined)  **\_\_weakref\_\_** = <attribute '\_\_weakref\_\_' of 'Smarts' objects> list of weak references to the object (if defined) |

|  |  |  |
| --- | --- | --- |
| **Functions** | | |
|  |  | **findbits**(fp, bitsperint)  Find which bits are set in a list/vector.     This function is used by the Fingerprint class.     >>> findbits([13, 71], 8)  [1, 3, 4, 9, 10, 11, 15]  **readfile**(format, filename)  Iterate over the molecules in a file.     Required parameters:     format     filename     You can access the first molecule in a file using:      mol = readfile("smi", "myfile.smi").next()        You can make a list of the molecules in a file using:      mols = [mol for mol in readfile("smi", "myfile.smi")]        You can iterate over the molecules in a file as shown in the  following code snippet...     >>> atomtotal = 0  >>> for mol in readfile("sdf","head.sdf"):  ...     atomtotal += len(mol.atoms)  ...  >>> print atomtotal  43  **readstring**(format, string)  Read in a molecule from a string.     Required parameters:     format     string     >>> input = "C1=CC=CS1"  >>> mymol = readstring("smi",input)  >>> len(mymol.atoms)  5 |

|  |  |  |
| --- | --- | --- |
| **Data** | | |
|  |  | **informats** = {'acr': 'ACR format', 'alc': 'Alchemy format', 'arc': 'Accelrys/MSI Biosym/Insight II CAR format', 'bgf': 'MSI BGF format', 'box': 'Dock 3.5 Box format', 'bs': 'Ball and Stick format', 'c3d1': 'Chem3D Cartesian 1 format', 'c3d2': 'Chem3D Cartesian 2 format', 'caccrt': 'Cacao Cartesian format', 'car': 'Accelrys/MSI Biosym/Insight II CAR format', ...}  **outformats** = {'alc': 'Alchemy format', 'bgf': 'MSI BGF format', 'box': 'Dock 3.5 Box format', 'bs': 'Ball and Stick format', 'c3d1': 'Chem3D Cartesian 1 format', 'c3d2': 'Chem3D Cartesian 2 format', 'cac': 'CAChe MolStruct format', 'caccrt': 'Cacao Cartesian format', 'cache': 'CAChe MolStruct format', 'cacint': 'Cacao Internal format', ...} |
